# Alterations of Lipid Metabolism in Cancer: Implications in Prognosis and Treatment

**DOI:** 10.3389/fonc.2020.577420

**Published:** 2020-10-28

**Authors:** Lara P. Fernández, Marta Gómez de Cedrón, Ana Ramírez de Molina

**Affiliations:** Precision Nutrition and Cancer Program, Molecular Oncology Group, IMDEA Food Institute, Campus of International Excellence (CEI) University Autonomous of Madrid (UAM) + CSIC, Madrid, Spain

**Keywords:** lipid metabolism, cancer prognosis, tumor microenviroment (TME), obesity, cancer risk, precision medicine, precision nutrition

## Abstract

Cancer remains the second leading cause of mortality worldwide. In the course of this multistage and multifactorial disease, a set of alterations takes place, with genetic and environmental factors modulating tumorigenesis and disease progression. Metabolic alterations of tumors are well-recognized and are considered as one of the hallmarks of cancer. Cancer cells adapt their metabolic competences in order to efficiently supply their novel demands of energy to sustain cell proliferation and metastasis. At present, there is a growing interest in understanding the metabolic switch that occurs during tumorigenesis. Together with the Warburg effect and the increased glutaminolysis, lipid metabolism has emerged as essential for tumor development and progression. Indeed, several investigations have demonstrated the consequences of lipid metabolism alterations in cell migration, invasion, and angiogenesis, three basic steps occurring during metastasis. In addition, obesity and associated metabolic alterations have been shown to augment the risk of cancer and to worsen its prognosis. Consequently, an extensive collection of tumorigenic steps has been shown to be modulated by lipid metabolism, not only affecting the growth of primary tumors, but also mediating progression and metastasis. Besides, key enzymes involved in lipid-metabolic pathways have been associated with cancer survival and have been proposed as prognosis biomarkers of cancer. In this review, we will analyze the impact of obesity and related tumor microenviroment alterations as modifiable risk factors in cancer, focusing on the lipid alterations co-occurring during tumorigenesis. The value of precision technologies and its application to target lipid metabolism in cancer will also be discussed. The degree to which lipid alterations, together with current therapies and intake of specific dietary components, affect risk of cancer is now under investigation, and innovative therapeutic or preventive applications must be explored.

## Introduction

Cancer is a significant public health problem and is the second leading cause of death globally (1). The World Health Organization (WHO) has indicated that lung, prostate, colorectal (CRC), stomach, and liver cancers are among the most frequent types of cancer in men, whereas breast, CRC, lung, cervical, and thyroid cancers are the most frequent among women. Together with the genetic alterations, environmental factors orchestrate the multifactorial and multistage characteristics of cancer, modulating the expression of both tumor suppressor genes and oncogenes.

One of the hallmarks of cancer is the abnormal regulation of cellular metabolism (2). Tumor cells exhibit high rates of aerobic glycolysis and an increased anabolism to support growth, proliferation, and survival. Consequently, metabolism-related pathways have acquired enormous relevance in cancer research. Together with the Warburg effect and the increased glutaminolysis, lipid metabolism plays a key role in cancer metabolic reprogramming (3). Lipids, a highly diverse class of biological molecules, exert three main functions in the cells. First, they are employed for energy storage, principally as triacylglycerol esters and steryl esters, in lipid droplets (LDs). In addition, lipids are structural components of cellular membranes, and they also operate as metabolic signaling messengers (4). The sterol regulatory element-binding proteins (SREBPs) are transcription factors that coordinate and regulate the synthesis of lipids. They act in response to upstream signaling networks and to the intracellular nutrient status, to regulate the expression of enzymes involved in cholesterol and fatty acid (FA) synthesis and uptake (5).

Together with genetic alterations mediating the metabolic reprogramming in a cell autonomous manner, cancer progression and dissemination also depend on the availability of nutrients and oxygen at the tumor microenvironment. Tumors communicate with the surrounding microenvironment, which includes fibroblasts, adipocytes, immune cells, endothelial cells, and components of the extracellular matrix—to support cancer proliferation and dissemination (6).

Furthermore, key lipid metabolism genes have been proposed as prognostic biomarkers in several types of cancer associated with tumor recurrence and/or survival (7, 8). Indeed, the role of lipid metabolism alterations in tumor cell migration, invasion, and angiogenesis has been clearly demonstrated (9–11).

The technical improvement and development of “omics” approaches, together with the availability of large public accessible databases, have redefined current strategies of cancer research (12) allowing to reanalyze, recapitulate, and update our knowledge of the relevance of lipid metabolism–related genes in cancer. Genomics and transcriptomics are being applied for precision medicine purposes in cancer. The design, validation, and use of polygenetic scores open a window of new opportunities to integrate “omics” technologies into clinical advice. Moreover, proteomics, metabolomics, lipidomics, and metagenomics will complete the full scenario (13). Additionally, clinical trials combining current chemotherapies with natural bioactive compounds toward altered lipid metabolism represent a promising strategy to improve cancer treatment (14).

In this review, we will discuss about the role of lipid metabolism alterations in cancer. We will explore their mechanism of action and their oncologic implications. Moreover, we will analyze current reports and knowledge of lipid metabolism biomarkers in the most frequent types of cancer. Finally, we will investigate their emergent use in precision medicine and precision nutrition strategies.

## Impact of Obesity in Cancer

In recent years, it has demonstrated that cancer malignancy not only relays on the genetic factors—oncogenic and tumor suppressor alterations—from patients, but also on environmental factors associated with lifestyle (15). In this regard, it has been shown that up to one-third of cancer deaths could be prevented by modifying environmental factors related to lifestyle such as physical activity and diet, alcohol consumption, and smoking. Unhealthy diets—high consumption of saturated FAs or high-glucose-content beverages—are also associated with the development of systemic metabolic alterations including obesity, insulin resistance, and metabolic syndrome, among others. Obesity, which is defined as a high body weight with excessive adipose tissue accumulation, can be considered as a chronic, multifactorial, and proinflammatory disease (6, 16). Obesity is a risk factor for several chronic diseases including type 2 diabetes mellitus, cardiovascular diseases, hepatic steatosis, and cancer initiation and progression (17, 18). In fact, the overall risk of cancer death is around 1.5- to 1.6-fold in individuals with a body mass index higher than 40 kg/m^2^ (19). The main types of cancer where obesity has been found associated with are prostate cancer (20), postmenstrual endometrial (21), breast cancer (22), ovary (23), bladder (24), liver (25), colon (26), and pancreas (22). During obesity, adipocytes accumulate in locations not classically associated with adipose tissue. Fat accumulation in ectopic sites is classified as central adipose tissue with systemic effects and locally accumulated adipose tissue supporting tumor microenvironment. The central adipose tissue leads to alterations in the levels of steroidal sex hormones, decreased insulin sensitivity, and low-grade inflammation (27), and it has been associated mainly with CRC (27) and breast cancer (6, 28). In addition, visceral depots of adipose tissue may provoke alterations in the cellular composition of cells surrounding the tumor microenvironment contributing to tumor cell proliferation and dissemination such as in the case of tumors located close to adipose tissues, such as breast, ovary, or colon tumors (6, 29).

The effects of tumor cells at the tumor microenvironment has been also found to associate with drug resistance (30). Cancer-associated adipocytes present metabolic features that sustain tumor progression and dissemination, because of the release of FAs and proinflammatory mediators, which contribute to support the surrounding tumor microenvironment (6). Thus, ovarian cancer partially relies on lipids provided by adipocytes at the tumor microenvironment (29, 31). Moreover, the hyperplasia and hypertrophy of adipose tissue diminish the levels of oxygen available, promoting angiogenesis, which may contribute to tumor dissemination (32). In this regard, breast, gastric, and colon cancers preferentially grow in adipocyte-enriched environments. In addition, excess of adipose tissue induces low chronic inflammation augmenting the circulating levels of proinflammatory interleukins (IL-6 and IL-8), tumor necrosis factor α, vascular endothelial growth factor (VEGF), and prostaglandins and leukotrienes, which have protumorigenic effects. Arachidonic acid (AA) is the main precursor of proinflammatory lipid mediators, such as prostaglandins, thromboxanes, and leukotrienes, which promote proliferation, cell survival, and dissemination of cancer cells. Inflammatory prostaglandins, such as prostaglandin E_2_ produced by COX2 (cyclooxygenase 2), activate epidermal growth factor receptor cell signaling to promote angiogenesis and the expression of matrix metalloproteases in colon cancer (33). Prostaglandins have been shown to inhibit the antitumor immune response by diminishing the activation of cytotoxic CD8^+^ T lymphocytes and the infiltration of natural killer cells and dendritic cells to the tumor (34). In this regard, COX2 inhibitors have been demonstrated to augment the response to immune checkpoint inhibitors in melanomas (35, 36).

In addition, it has been described that obese individuals present an altered gut microbiota and disrupted intestinal epithelium barrier. Dysbiosis is associated with microbial diversity together with an increase in proinflammatory species. Intestinal dysbiosis has been associated with gastric, CRC, and esophageal cancers (37, 38). Thus, the design of microbiota-targeting therapies is now considered as a feasible strategy in the clinic.

**Figure 1:**
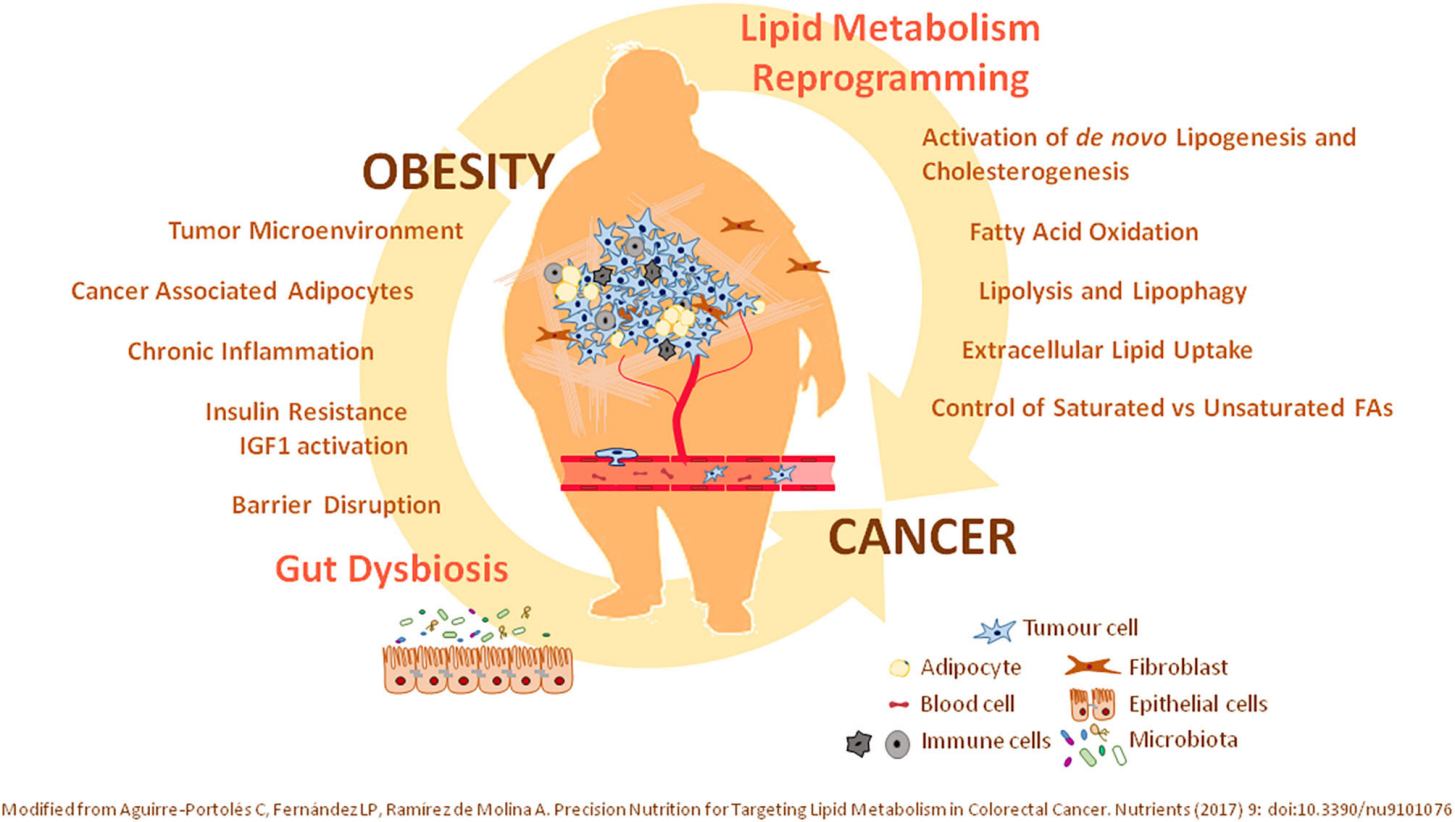
Relevance of lipid metabolism alterations in cancer. Illustrated is the crucial role of (i) oncogenic mutations supporting the lipid metabolism reprogramming in cancer, together with (ii) systemic lipid metabolic alterations associated with obesity—as an environmental modifiable risk factor. Precision interventions should include therapeutic clinical drugs targeting identified lipid metabolism molecular targets together with nutritional interventions—bioactive compounds, diet-derived ingredients—considering the nutritional and metabolic status of patients. T2DM, type 2 diabetes mellitus; IR, Insulin Resistance; TME, tumor microenviroment; CAAs, cancer-associated adipocytes; FAO, fatty acid oxidation; FA, fatty acid.

Because of the important metabolic link between obesity and the tumorigenic process (Figure 1), effective control of the nutritional and metabolic status of individuals (control of glucose, lipid levels, blood pressure, and chronic inflammation) might represent a specific and mechanistic approach to prevent and/or ameliorate cancer progression. In this scenario, precision nutrition has emerged as a complementary therapeutic tool in the management of metabolic alterations associated with cancer prognosis. Personalized nutrition compiles nutrigenetics (genetic variants and epigenetic signatures), deep phenotyping, and a wide spectrum of data concerning metabolic personalization through omics technologies—transcriptomics, metabolomics, lipidomics, and metagenomics. Importantly, nutritional interventions based on the knowledge of how nutrients and bioactive dietary compounds interact with the genome, metabolism, microbiome, etc., at the molecular level, represent an effective tool to fight against metabolic alterations.

## Lipid Metabolic Reprogramming of Oncogenic Pathways in Cancer

Cancer cells present metabolic alterations to provide the additional requirements of energy and metabolites for cancer cell proliferation and dissemination (2). Enormeous diversity exists between the different types of cancer, and even within the same tumor. Moreover, cancer cells are characterized by the continuous capacity to adapt to changes in the levels of nutrients and oxygen at the tumor microenvironment (6). The altered tumor metabolism depends not only on the cell autonomous genetic alterations, but also on additional factors including diet, food behavior, exercise, and microbiome. All these factors together will determine the biology of the developing tumor (39) (Figure 1).

One of the most frequent metabolic alterations observed in cancer is the increased of the glycolytic pathway, independently of the oxygen levels (Warburg effect) (40). Aerobic glycolysis in cancer is coupled to increase glutamine metabolism for the anaplerosis of intermediated of the tricarboxylic acid (TCA) cycle (41). In addition, different studies including *in vitro*, preclinical, and clinical trials have demonstrated the relevance of lipid metabolism to sustain cancer initiation and progression (6). The inhibition of lipid metabolic enzymes has been shown to induce tumor regression, to inhibit the metastatic spread, and/or to avoid drug resistance. Lipids not only are structural components of biological membranes, but also provide energy by means of β-FA oxidation (β-FAO), control the redox homeostasis, and act as signaling molecules affecting a plethora of crucial processes in cancer including proliferation, migration, invasion, transformation, tumor microenvironment reshaping, and/or modulation of inflammation (42). Cholesterol is a key component of the cell membranes affecting its fluidity, stabilizing specific areas (lipid rafts) to transduce intracellular cell signaling pathways (43), and being precursor of steroidal hormones (44). In addition, lipids are also signaling molecules such as proinflammatory prostaglandins or tromboxanes—synthesized from omega-6 AA (45), or anti-inflammatory omega-3 eicosapentaenoic acid (EPA) and docosahexaenoic acid, which availability depends on lipids provided from diet.

Herein, we describe potential strategies to target the altered lipid metabolism in cancer. In addition, as the uptake of high levels of saturated FAs from diet is a risk factor in several types of cancers, strategies to diminish lipolysis and promotion of healthy diets should also be considered.

### Activation of *de novo* Lipogenesis and Cholesterogenesis

Lipid metabolism alterations affect not only tumor cell proliferation, but also dissemination and resistance to chemotherapeutic drugs (46). Most of adult tissues obtain FAs, cholesterol, and lipids from diet; meanwhile, *de novo* synthesis of FAs and cholesterol is restricted to the liver and adipocytes. Tumors frequently present the capability to activate the *de novo* synthesis of cholesterol and FAs (47) making them more independent from externally provided lipids (48, 49). Importantly, targeting enzymes associated with *de novo* lipogenesis and/or the mevalonate pathway has been shown to inhibit tumor growth (6, 50).

**Figure 2:**
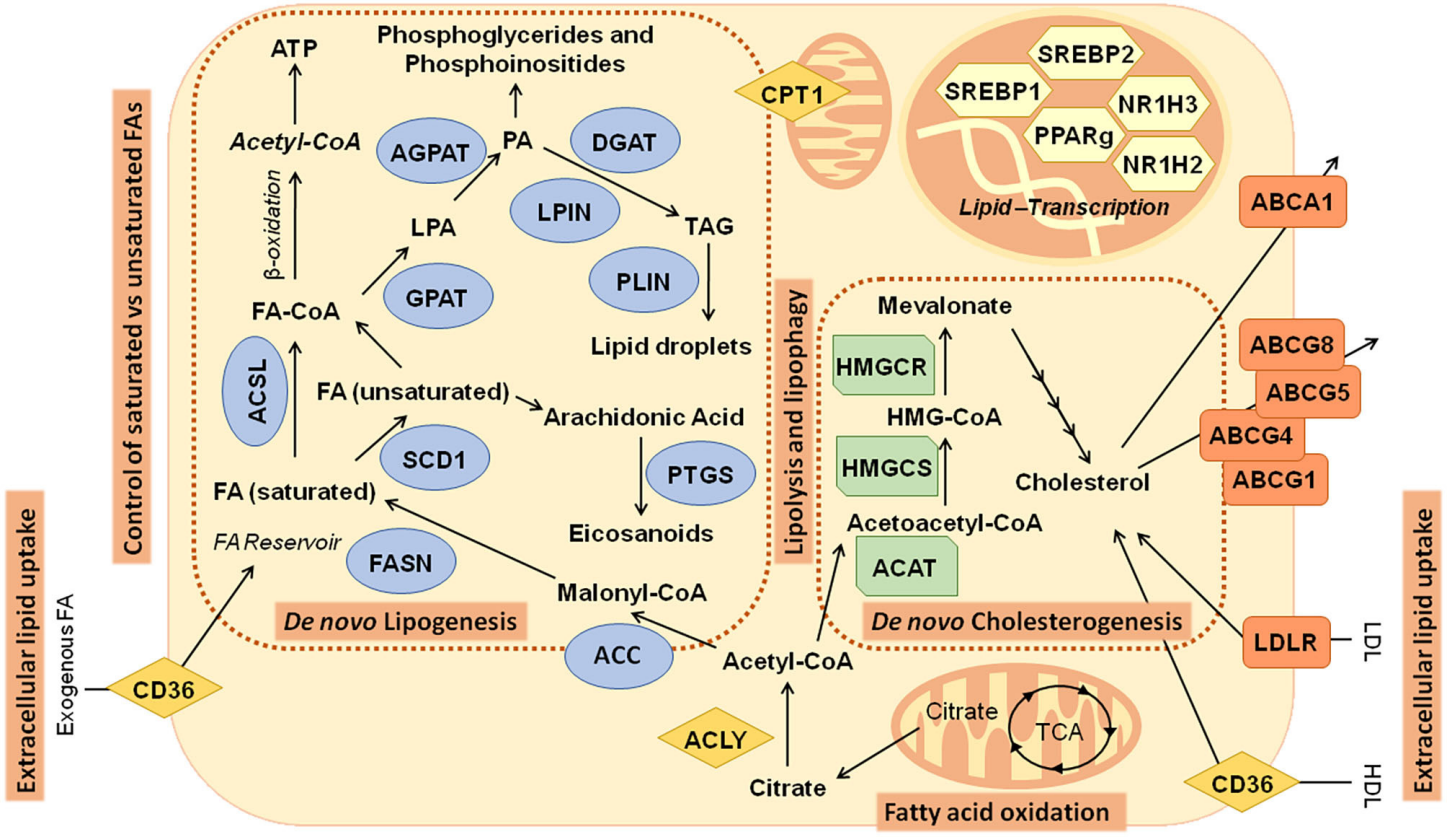
Main metabolic pathways related to lipid metabolism in cancer: Illustration of pathways and genes implicated in *de novo* lipogenesis—fatty acids and cholesterol biosynthesis. ABCA1, ATP-binding cassette subfamily A member 1; ABCG1, ATP-binding cassette subfamily G member 1; ABCG4, ATP-binding cassette subfamily G member 4; ABCG5, ATP-binding cassette subfamily G member 5; ABCG8, ATP-binding cassette subfamily G member 8; ACAT, acetyl-CoA acetyltransferase; ACC, acetyl- CoA carboxylase; ACLY, ATP citrate lyase; ACSL, acyl-CoA synthetase long chain; AGPAT, 1-acylglycerol-3-phosphate *O*-acyltransferase; CD36, CD36 molecule; CPT1, carnitine palmitoyltransferase; DGAT, diacylglycerol *O*-acyltransferase; FA, Fatty acids; FASN, fatty acid synthase; GPAT, glycerol-3-phosphate acyltransferase; HDL, high-density lipoprotein; HMGCR: 3-hydroxy-3-methylglutaryl-CoA reductase; HMGCS, 3-hydroxy-3-methylglutaryl-CoA synthase; LDL, low-density lipoprotein; LDLR, low-density lipoprotein receptor; LPIN, Lipin; NR1H2, nuclear receptor subfamily 1 group H member 2; NR1H3, nuclear receptor subfamily 1 group H member 3; PLIN, perilipin; PPARγ, peroxisome proliferator-activated receptor γ; PTGS, prostaglandin-endoperoxide synthase; SCD1, stearoyl-CoA desaturase; SREBP1, Sterol regulatory element binding transcription factor 1; SREBP2, sterol regulatory element binding transcription factor 2; TCA, tricarboxylic acid cycle.

FAs are synthesized from cytoplasmic acetyl-CoA (AcCoA), generated from citrate produced from glucose, glutamine, or acetate (48). ATP-citrate lyase (ACLY) generates AcCoA and oxaloacetate (OAA) from citrate (48, 51). AcCoA carboxylases (ACC1/2) carboxylase AcCoA to form malonyl-CoA. Subsequent condensation steps, catalyzed by FA synthase (FASN), forms the 16-carbon saturated FA palmitate. Palmitate is then elongated by FA elongases (ELOVL) and desaturated by stearoyl-CoA desaturase (*SCD1*) or FA desaturases (FADS) to form other nonessential FAs, such as the 18-carbon monounsaturated FA (MUFA) oleate (C18:1) (Figure 2).

Many enzymes implicated in *de novo* synthesis of FAs and cholesterol have been proposed as biomarkers for prognosis in specific types of cancer. FASN is found upregulated in prostate and breast cancer (47, 52), and ACLY has been shown to support tumor formation and transformation (51). Inhibition of several enzymes of *de novo* lipogenesis, such as FASN, and ACC1 and ACC2, has been tested in different cancer models showing their relevance on tumor growth inhibition (53).

Similarly, inhibition of hydroxymethylglutaryl-CoA (HMGCoA) reductase (HMGCR), by statins, leads to inhibition of cell proliferation of breast cancer cells (54) and tumor regression in several preclinical mouse models, and it is being tested in clinical trials (43). The overexpression of enzymes of the mevalonate pathway has been proposed as biomarkers of poor prognosis in breast cancer (55). Cholesterol is generated by the mevalonate pathway, by condensation of two AcCoA molecules to form 3-HMGCoA, which is then reduced to form mevalonate, and then isoprenoid farnesyl-pyrophosphate. Several studies have shown that targeting the synthesis of cholesterol inhibits cancer cell proliferation and transformation (56).

*De novo* synthesis of FAs and cholesterogenesis are transcriptionally regulated by SREBPs, which are downstream oncogenic pathways including PI3K/Akt (57) and c-Myc (47) (Figure 2).

The SREBP family includes three transcription factors: SREBP1a and SREBP1c, which are derived from *SREBF1* gene by alternative splicing (58), and SREBP2, which is encoded by *SREBF2* gene. SREBPs are bound to the endoplasmic reticulum (ER) as inactive precursors (59). When the intracellular levels of cholesterol are high, insulin-induced genes interact with SREBP-cleavage–activating proteins (SCAPs) to retain SREBP inactive precursors attached to the ER. When cholesterol levels are low, SCAPs facilitate the translocation SREBPs to the Golgi apparatus to be further processed releasing the active forms (56). SREBP1 promotes the expression of lipogenic genes; meanwhile, SREBP2 regulates the expression of genes involved in the synthesis, uptake, and efflux of cholesterol. Nevertheless, SREBP1 and SREBP2 have overlapping activities. Both SREBP1 and SREBP2 are found overexpressed in several cancers. Regulation of the intracellular content of cholesterol has also been shown crucial for cancer cell survival. The ATP-binding cassette transporter (ABCA1) controls the efflux of cholesterol to ApoA-coated lipoproteins (57). Recently, it has been demonstrated that activation of p53 increases the retrograde transport of cholesterol from the plasma membrane to the ER, to prevent SREBP2 maturation (60). In addition, cholesterol levels are fine tune regulated by microRNA33—encoded by an intron within the *SREBF2* gene (51)—which targets ABCA1. In addition, the esterification of cholesterol for storage in LDs, by sterol *O*-acyltransferase 1 (ACAT1), has been shown to augment the survival in prostate cancer (61).

### Fatty Acid Oxidation in Cancer

In addition to *de novo* synthesis of FAs and cholesterol, the mobilization of intracellular FAs for FAO at mitochondria is crucial for cancer survival and dissemination. It is well-known that tumor cells present higher levels of reactive oxygen species (ROS) than not tumor cells, which allow them to activate prosurvival and epithelial-to-mesenchymal transition programs to support cancer progression and dissemination. Nevertheless, excessive ROS may promote apoptotic cell death. It has been demonstrated that enzymes implicated in the mobilization of intracellular neutral lipids provide metabolic flexibility to increase the levels of FAs for oxidation at mitochondria. In the FAO pathway, acyl-CoAs are cyclically dehydrogenated, hydrated, and decarboxylated, resulting in the progressive shortening of the FA, together with the production of NADH and FADH_2_ and AcCoA. NADH and FADH_2_ will be used for ATP production in the electron transport chain, and AcCoA can enter the Krebs cycle. AcCoA together with OAA gives rise to citrate, which after being exported to cytoplasm, can enter two metabolic pathways to produce cytosolic NADPH (62).

Enhanced mitochondrial β-oxidation of FAs has been described in pancreatic cancer (63, 64) and in metastatic breast cancer (65). FAO not only provides energy when glucose becomes limiting, but it also contributes to a better control of the oxidative stress, by augmenting the intracellular levels of NADPH (66). Increased FAO augments survival in leukemia and gliomas by counteracting the metabolic oxidative stress. Moreover, FAO has been shown crucial for the survival of cells from solid tumors when undergoing loss of attachment, which triggers *anoikis* or cell death due to oxidative stress (67, 68).

In addition, FAO is also influenced by the tumor microenvironment such as in the case of ovarian cancers, which preferentially metastasizes to the omentum enriched in adipocytes, which provides lipids for ATP and NADPH production to control metabolic stress during metastasis.

### Regulation of FA Storage and Intracellular FA Mobilization (Lipolysis and Lipophagy)

*De novo* synthesis of FAs in cancer cells is coupled to additional processes to accommodate the increase in the intracellular lipid content, to preserve the homeostasis between lipid storage and lipid mobilization (69). FAs from *de novo* lipogenesis are accumulated into neutral lipids (stored in LDs) and phospholipids (in membranes). LDs are complex and dynamic organelles consisting of a neutral lipid core surrounded by a phospholipid monolayer and a complex proteome associated. LDs itself have been proposed as novel diagnostic biomarkers for glioblastoma. It has been demonstrated that while they are not detectable in low-grade gliomas or normal brain tissues, they are common in glioblastoma, the most lethal brain tumor (70). Among the LD-associated proteins, there are enzymes of the sterol biosynthetic pathway, the acyl-CoA metabolism (ACSLs), and triacylglycerol (TAG) biosynthesis. Structural proteins, such as perilipins (PLINs) or caveolins, are critical for the integrity of LDs to avoid collapse and to protect them from lipolysis (Figure 2). Cancer cells present higher amounts of LDs than normal cells (71). Increased expression of PLIN2 has been shown to favor the accumulation of LDs (72), contributing to a better control of the ER stress, to increase the protection against ROS, and to augment the resistance to therapeutic drugs in cancer cells. On the contrary, PLIN2 depletion significantly attenuated the proliferation of colon cancer cells (73), supporting the LD-associated proteins as potential druggable targets for cancer treatment (11).

The increase in *de novo* synthesis of FAs in cancer cells requires efficient and complementary lipolytic mechanisms to accommodate the intracellular lipid content. Thus, lipolysis allows the stored lipids to be available for the synthesis of phospholipids and lipid signaling mediators and/or to increase the levels of ATP or NADPH when required. Several enzymes involved in lipolysis—adipose TAG lipase (ATGL), hormone-sensitive lipase (HSL), monoacylglycerol lipase (MAGL)—have been described to promote tumorigenesis (74). In this sense, ATGL knockdown in HCT116 CRC cells reduced cell proliferation (75). Increased levels of MAGL are associated with aggressive cancer types such as melanoma and ovarian and breast cancer (74), and inhibition of MAGL suppresses cancer cell migration, invasion, and survival (76). Recently, it has been demonstrated that glioblastomas, which acquire large amounts of free FAs, upregulate diacylglycerol-acyltransferase 1 (DGAT1) to store the excess FAs into triglycerides and LDs (77). Inhibition of DGAT1 disrupted lipid homeostasis, resulting in increased levels of ROS leading to apoptotic cell death.

In addition, a specific function of autophagy associated with the regulation of the intracellular lipid content—lipophagy—has been described to augment resistance to cell death in cancer (78).

### Extracellular Lipid Uptake

In addition, similar to normal cells, cancer cells can uptake exogenous lipids when *de novo* lipogenesis is inhibited. Upregulation of cell surface receptors, such as cluster of differentiation 36 (CD36) (Figure 2), has been found to augment metastasis (79, 80). CD36 inhibition diminished tumor growth and metastasis in preclinical models of prostate cancer (80). Moreover, the expression of low-density lipoprotein receptor (LDLR) for the internalization of low-density lipoproteins (LDLs) has been found upregulated in renal cell carcinoma (RCC) cells (81). FA-binding proteins (FABPs) contribute to augment the lipid uptake, as well as the intracellular lipid trafficking in cancer cells (82). In breast cancer and glioblastoma cell lines, it has been shown that the uptake of extracellular FAs during hypoxia is sustained by the upregulation of FABP3 and FABP7; meanwhile, FABP5 increases cell proliferation and growth in prostate cancer (83).

### Control of Saturated vs. Unsaturated FAs

Depending on the source of FAs, *de novo* lipogenesis or extracellular lipid uptake, the levels of saturated FAs incorporated in the phospholipids of cell membranes are different. The lipogenic pathway increases the saturation level of cell membranes with saturated and MUFAs (84), which are less sensitive to suffer lipid peroxidation compared to polyunsaturated acyl chains (PUFAs) mainly obtained from diet. This way, *de novo* lipogenesis contributes to augment the resistance to oxidative stress and chemotherapy in cancer cells (85).

Nevertheless, excessive accumulation of saturated FAs in the cell membranes can lead to lipotoxicity. In this regard, SCD1 inhibition induces ER stress and apoptosis in cancer cells and diminishes the tumor growth in xenografts models of colon and lung cancers (86). During tumor growth, inner parts of the tumors are faced to hypoxia and reduced nutrient availability. Tumors have developed different strategies to balance the levels of saturated vs. unsaturated FAs. Thus, tumors anticipate lipotoxicity by augmenting the uptake of MUFAs/PUFAs from plasma, which are further stored into LDs or incorporated into phospholipids at the cell membranes. As SCD1 activity requires oxygen, during hypoxia some tumors rely on the activity of DGATs to incorporate MUFAs into TG, which are further accumulated into LDs (Figure 2). In addition, tumors balance, via the Lands cycle, the levels of saturated vs. unsaturated FAs in the phospholipids at the cell membranes. Recently, a process known as ferroptosis has been described associated with high levels of MUFA/PUFAs in the phospholipids of cell membranes, which induce cell death by means of their oxidation through the Fenton pathway. Long-chain FA acyl CoA synthetases (ACSLs)—implicated in the long chain FA activation—may control ferroptosis, as distinct isoforms use distinct substrates. Meanwhile, ACSL4 has PUFAS as main substrates such as AA, ACSL3 can activate both MUFAs and PUFAs, allowing a better control of the excessive accumulation of PUFAs in phospholipids (87). In addition, ACSL3 allows a better control of FA distribution between LD storage or β-FAO, providing a better control of the oxidative stress (42).

## Lipid Metabolism Alterations and Cancer Prognosis

Alterations of lipid metabolism genes are found in many tumor types, predominantly, but not exclusively, because lipid metabolism can modulate different cellular processes that go from plasmatic and organelle membrane organization and plasticity (88, 89), substrate supply for ATP synthesis, (62) and intracellular cell signaling activation (90). Cancer tissues display abnormal activation of *de novo* lipogenesis and cholesterogenesis (91). Extremely proliferative cancer cells exhibit an intense lipid and cholesterol avidity, which they satisfy by increasing the uptake of dietary or exogenous lipids and lipoproteins or activating lipogenesis or cholesterol synthesis (3). Importantly, this aberrant lipid metabolism does not only influence the primary tumor, but the exogenous lipids produced by tumor microenvironment can also influence malignancy (14, 92–95). Besides, three basic steps during metastasis: migration (96), invasion (9, 10) and angiogenesis (97, 98), are affected by lipid metabolism regulation (11).

Nowadays, there are increasing evidences of the role of lipid metabolism alterations as biomarkers of cancer prognosis and survival. Here, we are going to review previous knowledge on the prognostic value of lipid-related genes that belong to FAs and cholesterol pathways (Figure 2) in the most frequent types of cancer according to the WHO: lung, CRC, breast, and prostate.

Furthermore, “omics” data publicly available in huge searchable databases facilitate addressing specific medical issues in thousands of patients. Remarkably, The Cancer Genome Atlas (TCGA) gene expression dataset (https://www.cancer.gov/tcga) and The Human Protein Atlas website together with The Pathology Atlas online tool (https://www.proteinatlas.org/humanproteome/pathology), which contains mRNA data from TCGA study and protein expression data from different forms of human cancer (99–101), allowing us to obtain a global view of the putative implications of lipid metabolism–related genes in cancer prognosis. Data from TCGA visualized using The Pathology Atlas online tool, are summarized in Table 1.

**Table 1:** Prognostic value of lipid metabolism–related genes.

	**Literature**								**The Cancer Genome Atlas (TCGA)-The Protein Atlas**																
**Prognostic value**	**LC**		**CRC**		**BC**		**PC**		**LC**	**CRC**	**BC**	**PC**	**CC**	**EC**	**G**	**HNC**	**LC**	**M**	**OC**	**PC**	**RC**	**SC**	**TC**	**ThC**	**UC**
**Fatty acid–related pathways**																									
**Fatty acid synthesis**																									
ACLY		(61, 102, 103)		(104)																					
pACC		(105)		(39)																					
ACACA																									
ACACB				(106)																					
FASN		(107)		(36, 108–112)		(113–115)		(116–119)																	
ACSL1				(7–9, 120, 121)																					
ACSL3		(+)																							
ACSL4				(7–9, 120, 121)		(122)																			
ACSL5						(121, 123)																			
ACSL6																									
SCD1		(124)		(7, 125)		(126)																			
FADS1		(127)																							
FADS2																									
FADS3																									
FADS4																									
FADS6																									
FADS7																									
FADS8																									
PTGS1																									
PTGS2		(128–130)		(131)		(132)		(133)																	
GPAT1																									
GPAT2																									
GPAT3																									
GPAT4																									
AGPAT1		(7, 8)																							
AGPAT2																									
AGPAT3																									
AGPAT4																									
AGPAT5																									
LPIN1		(134)				(135)		(136)																	
LPIN2																									
LPIN3																									
PLIN1		(137)				(138)																			
PLIN2						(139)																			
PLIN3																									
PLIN4																									
PLIN5																									
DGAT1																									
DGAT2						(140)																			
**Fatty acids–related transportation**																									
CD36				(141)		(141)		(141)																	
CPT1A						(142, 143)																			
CPT1B																									
CPT1C																									
**Cholesterol-related pathways**																									
**Cholesterol synthesis**																									
ACAT1								(144, 145)																	
ACAT2																									
HMGCS1																									
HMGCS2				(146)				(147)																	
HMGCR				(148)		(149, 150)																			
**Cholesterol-related transportation**																									
ABCA1				(7, 8, 151)																					
ABCG1																									
ABCG4		(152)																							
ABCG5				(153)																					
ABCG8																									
LDLR				(154)																					
**Lipid transcription**																									
**Transcription factors**																									
SREBP1						(155)																			
SREBP2				(156)				(157)																	
PPARγ		(158, 159)		(160)		(161, 162)		(163)																	
NR1H2				(156)																					
NR1H3		(164)		(156)																					

*Favorable* 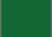

*Unfavorable* 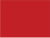

*Gene prognostic value reported in the literature in most frequent types of cancer according to the World Health Organization (WHO) together with gene prognostic value using data from The Cancer Genome Atlas (TCGA) and visualized using The Pathology Atlas online tool. Abbreviations: LC, lung cancer; CRC, colorectal cancer; BC, breast cancer; PC, prostate cancer; CC, cervical cancer; EC, endometrial cancer; G, glioma; HNC, head and neck cancer; LC, liver cancer; M, melanoma; OC, ovarian cancer; PC, pancreatic cancer; RN, renal cancer; SC, stomach cancer; TC, testis cancer; ThC, thyroid cancer; UC, urothelial cancer; ACLY, ATP citrate lyase; pACC, phospo acetyl-CoA carboxylase; ACACA, acetyl-CoA carboxylase A; ACACB, acetyl-CoA carboxylase B; FASN, fatty acid synthase; ACSL1, acyl-CoA synthetase long chain family member 1; ACSL3, acyl-CoA synthetase long chain family member 3; ACSL4, acyl-CoA synthetase long chain family member 4; ACSL5, acyl-CoA synthetase long chain family member 5; ACSL6, acyl-CoA synthetase long chain family member 6; SCD1, stearoyl-CoA desaturase1; FADS1, fatty acid desaturase 1; FADS2, fatty acid desaturase 2; FADS3, fatty acid desaturase 3; FADS4, fatty acid desaturase 4; FADS6, fatty acid desaturase 6; FADS7, fatty acid desaturase 7; FADS8, fatty acid desaturase 8; PTGS1, prostaglandin-endoperoxide synthase 1; PTGS2, prostaglandin-endoperoxide synthase 2; GPAT1, glycerol-3-phosphate acyltransferase 1; GPAT2, glycerol-3-phosphate acyltransferase 2; GPAT3, glycerol-3-phosphate acyltransferase 3; GPAT4, glycerol-3-phosphate acyltransferase 4; AGPAT1, 1-acylglycerol-3-phosphate O-acyltransferase 1; AGPAT2, 1-acylglycerol-3-phosphate O-acyltransferase 2; AGPAT3, 1-acylglycerol-3-phosphate O-acyltransferase 3; AGPAT4, 1-acylglycerol-3-phosphate O-acyltransferase 4; AGPAT5, 1-acylglycerol-3-phosphate O-acyltransferase 5; LPIN1, lipin 1; LPIN2, lipin 2; LPIN3, lipin 3; PLIN1, perilipin 1; PLIN2, perilipin 2; PLIN3, perilipin 3; PLIN4, perilipin 4; PLIN5, perilipin 5; DGAT1, diacylglycerol O-acyltransferase 1; DGAT2, diacylglycerol O-acyltransferase 2; CD36, CD36 molecule; CPT1A, carnitine palmitoyltransferase 1A; CPT1B, carnitine palmitoyltransferase 1B; CPT1C, carnitine palmitoyltransferase 1C; ACAT1, acetyl-CoA acetyltransferase 1; ACAT2, acetyl-CoA acetyltransferase 2; HMGCS1, 3-hydroxy-3-methylglutaryl-CoA synthase 1; HMGCS2, 3-hydroxy-3-methylglutaryl-CoA synthase 2; HMGCR, 3-hydroxy-3-methylglutaryl-CoA reductase; ABCA1, ATP-binding cassette subfamily A member 1; ABCG1, ATP-binding cassette subfamily G member 1; ABCG4, ATP-binding cassette subfamily G member 4; ABCG5, ATP-binding cassette subfamily G member 5; ABCG8, ATP-binding cassette subfamily G member 8; LDLR, low-density lipoprotein receptor; SREBP1, Sterol regulatory element binding transcription factor 1; SREBP2, Sterol regulatory element binding transcription factor 2; PPARγ, peroxisome proliferator-activated receptor γ; NR1H2, nuclear receptor subfamily 1 group H member 2; NR1H3, nuclear receptor subfamily 1 group H member 3. (+) Unpublished results*.

### Fatty Acid–Related Alterations as Biomarkers of Cancer Prognosis and Survival

*De novo* FA biosynthesis occurs in the cellular cytoplasm. FAs originate from acetyl-coenzyme A, which is mostly provided by citrate produced by the TCA cycle. Switch of citrate into AcCoA is catalyzed by ATP citrate lyase (ACLY) (Figure 2). Consequently, ACLY is a key enzyme connecting carbohydrate to lipid metabolism by producing AcCoA from citrate for both FA and cholesterol synthesis (61). Several studies have associated ACLY expression in tumor tissues with worse prognosis. ACLY overexpression correlated with stage, differentiation grade, and a poorer prognosis in non–small cell lung cancer (NSCLC) (61). Besides, in combination with the glucose transporter *GLUT1, ACLY* was also an independent prognostic factor for overall survival (OS) in node-negative patients with NSCLC (102). However, one study reports that young NSCLC patients overexpressing *ACLY* had longer OS, in contrast to older patients where overexpression of ACLY appears to predict the opposite prognosis (103). *ACLY* also facilitates colon cancer cell metastasis, and high expression levels of *ACLY* and Catenin β1 (*CTNNB1*) protein were positively correlated with metastasis of colon cancer (104). Data from TCGA showed *ACLY* as a putative unfavorable marker of cervical and liver cancer (Table 1).

At the genomic level, single nucleotide polymorphisms (SNPs) in *ACLY* gene have been described as independent cancer prognostic markers in Asiatic populations. SNP rs9912300 in *ACLY* gene was significantly associated with OS in lung cancer patients (165). rs9912300 and rs2304497, both functional *ACLY* SNPs, exhibited a significant association with risks of death and recurrence in patients with advanced stages of colon cancer (166).

The following step of FA biosynthesis involves the activation of AcCoA to malonyl-CoA, which is catalyzed by AcCoA carboxylase (ACC) (Figure 2). ACC is a complex multifunctional enzyme system. There are two ACC forms, α (ACACA) and β (ACACB), encoded by two different genes. High phospho-acetylCoA carboxylase (pACC) was an independent marker for prediction of better survival in lung adenocarcinoma patients (105), and low pACC levels detected by immunohistochemistry were associated both with worse OS and progression-free survival in advanced stage CRC (167). In the same line, gene expression analysis reported that patients with upregulation of six of these hub genes (genes with high correlation in candidate modules) (*ACACB*, acyl-CoA dehydrogenase medium chain, adiponectin, *C1Q* and collagen domain containing, acyl-CoA synthetase short-chain family member 2, phosphoenolpyruvate carboxykinase 1 and PLIN1) displayed improved breast cancer prognosis (106). In TCGA dataset, *ACACA* gene expression is an unfavorable risk factor for liver cancer, whereas *ACACB* is a favorable prognostic factor for both renal and pancreas tumors (Table 1). Finally, it has been described in prostate cancer that genetic alterations of *ACACA, FASN*, and *SREBF1* predicted worse overall patient survival (168).

Malonyl-CoA is coupled to the multifunctional enzyme FASN. Repeated cycles of acetyl group's condensation produce the primary FA palmitate that can suffer separate elongation and/or unsaturation cycles to yield other FA molecules (169) (Figure 2). FASN is the key enzyme necessary for the *de novo* synthesis of long-chain FAs. FASN has been found overexpressed in nearly all of cancer tissues, and its expression is associated with a poorer prognosis.

One study reported that *FASN* gene expression was higher in the adjacent non-cancer tissue than in the NSCLC tissue, but authors concluded that it was a weaker predictor of shorter patient survival (170). However, a correlation analysis between expression levels of CD276 (B7-H3) and FASN exhibited a positive correlation with poor prognosis in clinical lung cancer tissues (107).

FASN levels were clearly upregulated in CRC tissues with high expression of FASN significantly associated with lymph node metastasis (108), liver metastasis (109), TNM (tumor, node, metastasis) stage, and poor prognosis (36). Moreover, a significant association was shown between FASN and VEGF expression, suggesting the involvement of FAS in tumor angiogenesis (110). Interestingly, one study reported that, among non-obese patients with colon cancer, tumoral FASN overexpression is associated with better survival, while among moderately overweight or obese patients, FASN overexpression may predict a poorer outcome (111). Furthermore, a panel of five genes including *FASN* (*ACOT8/ACSL5/FASN/HMGBCS2/SCD1*) has been reported to display a improved prognostic performance than validated clinical risk scales, and it is applicable for early discovery of CRC and tumor recurrence (112). Finally, FASN levels in serum were also examined in CRC patients, where it was associated with tumor stage (171), and high FASN levels are considered as a promising independent predictor of CRC with advanced phases, late clinical stages, and shorter survival (172).

FASN is associated with poor prognosis in breast and prostate cancer, and its inhibition is selectively cytotoxic to human cancer cells (113). FASN was found overexpressed in most of the triple-negative breast cancer (TNBC) patients but not always correlated with OS or disease-free survival. High FASN was significantly associated with positive node status (114). A greater part of clinically HER2-positive tumors was achieved as FASN overexpressors. Reclassification of HER2-positive breast tumors based on FASN gene expression predicted a significantly inferior relapse-free and distant metastasis-free survival in HER2^+^/FASN^+^ patients (115).

A substantial subset of prostatic cancers displays clearly elevated expression of immunohistochemically detectable FASN, a feature that has been associated with poorer prognosis (116–119). Furthermore, high expression level of FASN resulted in a significantly poor prognosis of pancreatic cancer (173), and data from TCGA study suggest that *FASN* expression could be a marker of bad outcome in cervical and renal cancer (Table 1).

In addition, several genetic changes in *FASN* gene have been associated with cancer prognosis. Two SNPs rs4246444 and rs4485435 were significantly associated with the recurrence of NSCLC (165). Finally, as it has been previously mentioned in prostate cancer that genetic alterations of *FASN* together with *ACACA* and *SREBF1* predicted worse prognosis (168).

Then, FAs are activated with CoA by fatty acyl-CoA synthetases (ACSLs) (Figure 2), which is essential for phospholipid and triglyceride synthesis and lipid modification of proteins in addition to for FA β-oxidation (169).

Family of long-chain acyl-CoA synthetases has been extensively proposed as putative prognostic biomarkers of cancer. *ACSL3* is up-regulated in lung cancer compared to the healthy lung tissue (174), and recently, an association with *ACSL3* expression, NSCLC prognosis, and the efficacy of statins treatment has been discovered (L. P. Fernandez et al., unpublished results). *ACSL3* was also found to be overexpressed in estrogen receptor–negative breast cancer (175) and prostate cancer (176). *ACSL1* and *ACSL4* overexpression was associated with a poor clinical outcome in stage II CRC patients (7–9, 120, 121). In addition, *ACSL4* is considered a biomarker for liver and breast cancers (122, 177). By contrast, downregulation of *ACSL5* in breast cancer was associated with a poorer prognosis (121, 123). There have not been reported associations between *ACSL6* and cancer survival (178).

An *in silico* study (121) also suggested that high *ACSL1* expression was associated with worse outcome in lung cancer patients, and *ACSL3* overexpression was associated with worse survival in patients with melanoma. In contrast, high *ACSL3* expression predicted a better prognosis in ovarian cancer. In the same study, *ACSL4* overexpression predicted bad prognosis in CRC, but good prognosis in breast, brain, and lung cancers. High expression of *ACSL5* predicted good prognosis in breast, ovarian, and lung cancers. Finally, low *ACSL6* predicted a worse prognosis in acute myeloid leukemia. *In silico* analysis of TCGA data (Table 1) suggested that *ACSL1, ACSL4*, and *ACSL5* are associated with favorable outcome in renal, urothelial, and endometrial cancers, respectively, whereas *ACSL3* expression predicts poor survival in lung and liver tumors.

Genetically, a 3′-UTR polymorphism in *ACSL1* is associated with *ACSL1* expression levels and poor clinical outcome in CRC patients (14, 120). Patients carrying the *ACSL1* rs8086 T/T genotype had significantly reduced disease-free survival compared with patients carrying the C/T or C/C genotype, with 3-fold higher risk of recurrences. T/T genotype for rs8086 is correlated with worse clinical outcome and simultaneously associates with high *ACSL1* mRNA levels (14, 120).

Stearoyl CoA desaturase 1 (*SCD1*) catalyzes the rate-limiting step in the synthesis of MUFAs that are the main components of tissue lipids. *SCD1* has been associated with tumor development, late stage, and reduced survival in lung adenocarcinoma (124). Together with other three lipid metabolism–related genes (*ABCA1, ACSL1*, and *AGPAT1*), *SCD1* expression separated stage II colon cancer patients with a 5-fold higher risk of relapse (7). Moreover, positive associations between *SCD1* expression and CRC patient clinical status and the expression of cancer stem cell–related genes (*WNT* and *NOTCH* signaling) were found based on TCGA data analysis (125). In the same line, high *SCD1* expression is associated with shorter survival in breast cancer patients (126). Table 1 shows that *SCD1* is an unfavorable marker of survival in renal and urothelial cancer in TCGA tumors. Other desaturases have also been analyzed as prognostic markers, and, for example, reduced expression of *FADS1* suggests pessimistic prognosis for NSCLC patients (127).

Glycerol-3-phosphate acyltransferase (GPAT) catalyzes the first step in the production of almost all membrane phospholipids. GPAT transfers an acyl group from acyl-CoA or acyl-ACP at the sn-1 or-2 position of glycerol 3-phosphate originating lysophosphatidic acids (LPAs) (179). LPA is a substrate for synthesis of numerous important glycerolipid intermediates, such as storage lipids, extracellular lipid polyesters, and membrane lipids (Figure 2). Four GPATs have been discovered; nevertheless, only GPAT1 (GPAM) has been related to cancer outcome. High GPAT1 expression has been associated with reduced OS in ovarian cancer (180). Data from TCGA suggested that *GPAT1* could be a favorable prognostic marker in renal cancer, while *GPAT3* is a putative biomarker of good prognosis in renal cancer in contrast to urothelial cancer. Finally, *GPAT4* expression could have a risk effect in ovarian and endometrial cancers, and a protective one in prostate and urothelial cancer (Table 1).

LPA is further metabolized to phosphatidic acid (PA) by AGPATs (1-acylglycerol-3-phosphate *O*-acyltransferases) (Figure 2). AGPAT1 belongs to previously mentioned transcriptional signature where combined analysis of four genes, *ABCA1, ACSL1, AGPAT1*, and *SCD1*, is associated with higher risk of relapse in stage II CRC patients (7). Furthermore, individuals with upregulation of *AGPAT1* expression have an increased risk of CRC recurrence, independently of tumor stage (8). Expression of *AGPAT2* was significantly related to decreased OS as well as to shorter progression-free survival in ovarian cancer patients younger than 60 years (181). When we consider tumors from TCGA study, several associations were found (Table 1). *AGPAT3* is a marker of good prognosis in renal cancer and predicts bad outcome in cervical cancer. High expression levels of *AGPAT4* may be associated with poor prognosis in cervical and renal cancers, whereas *AGPAT5* is an unfavorable prognostic marker in liver cancer and a favorable one in CRC.

Then PA is converted to diacylglycerol (DAG) by LPIN, a PA phosphatase. Three LPIN isoforms have been described. LPIN1 is upregulated in lung adenocarcinoma tumor tissues, and high LPIN1 expression was correlated with poor prognosis of patients with lung adenocarcinoma (134). In breast cancer, previous results seem to indicate that the high LPIN expression is related to a good prognosis (135). However, in basal-like TNBC, high LPIN1 expression correlates with the poor prognosis of these patients (136). In TCGA dataset analysis, *LPIN2* appears as a favorable prognostic marker in head and neck cancers, while *LPIN3* could be an unfavorable biomarker of endometrial, ovarian, and renal tumors (Table 1).

The final step in triacylglycerols synthesis is catalyzed by DGAT, which esterifies the DAG with a FA. Two human DGAT isoforms have been described (182). The expression of DGAT2 in HER2-positive breast cancer was decreased and was closely related to patient prognosis (140). However, data from TCGA reported *DGAT2* as an unfavorable prognostic factor for endometrial cancer (Table 1).

Subsequently, TAGs could be stored in LDs, and PLINs, an LD surface family of proteins, are necessary for optimal lipid storage and FA release. There are multiple PLIN proteins encoded by mRNA splice variants of a single PLIN gene. PLIN1 expression in lung adenocarcinoma is associated with apocrine-like features and poor clinical prognosis (137). In contrast, *PLIN1* mRNA expression is significantly downregulated in human breast cancer. The reduced expression of PLIN1 is an independent predictor of OS in estrogen receptor–positive and luminal A-subtype breast cancer patients (138). Also in breast cancer, low expression of PLIN2 was associated with favorable prognosis (139). The prognostic effects of PLINs in several types of cancer from TCGA analysis are multiple and very diverse (Table 1).

Eicosanoids are biologically active metabolites of AA and are produced by cyclooxygenases 1 and 2 (COX1 and COX2) [also known as prostaglandin-endoperoxide synthase 1 and 2 (PTGS1 and PTGS2)]. They are overexpressed in a variety of malignant tumors. It has been reported that the mRNA levels of *COX-1* and *COX-2* in lung cancer patients were significantly higher than in normal patients (183). However, another study reports that in tumor cells COX-2 rather than COX-1 expression may account for the variable prostanoid production seen in NSCLC (128). It is clear that multivariate analysis showed that tumoral *COX-2* mRNA expression and lymph node status were the most important independent prognostic predictors for NSCLC survival and disease relapse (129). Elevated COX-2 expression in tumors was significantly associated with lower survival in NSCLC and might be useful in identifying patients who would benefit from additional therapies for managing their disease (130).

The same tendency was observed in CRC, where elevated COX-2 expression, but not that of COX-1, was significantly associated with reduced survival and recognized as an independent prognostic factor (131). However, it has been reported that COX-1 and COX-2 expression is highly variable in Dukes' C tumors, and changes in COX-1 expression may be of importance in CRC (184).

COX-2 expression level and its prognostic value are also a matter of debate in breast cancer (185). Nevertheless, at least eight immunohistochemical reports have explored expression of COX-2 in a total of 2,392 primary breast carcinomas, of which 40% were found to be COX-2 positive (132). At least, four studies have detected that overexpression of COX-2 is linked to poor prognosis in breast cancer. These studies provide the basis for further estimation of a possible therapeutic effect of COX inhibitors in therapy of breast cancer.

In prostate cancer, a subset of Chinese patients with high-COX-2 expression showed minor disease-free and OS rates than those with low COX-2 expression. In this work, univariate and multivariate analyses suggested that the status of COX-2 protein expression was an independent prognostic factor for patients' survival (133).

Data from TCGA showed *COX-1* and *COX-2* as unfavorable markers of renal cancer, whereas only *COX-1* was a risk biomarker of urothelial cancer (Table 1).

Chronic inflammation is a recognized risk factor for CRC, and polymorphisms in genes regulating inflammatory processes appear to modify the risk of neoplasia and the efficacy of non-steroidal anti-inflammatory drugs in CRC chemoprevention. *COX-1* polymorphism G213G was significantly associated with an increased CRC (186). Finally, another study reports four *COX-1* variants that were associated with CRC survival. rs1213266 was associated with approximately 50% lower CRC mortality. Three other variants, including L237M, resulted in significantly elevated CRC mortality risk (187).

Proteins related to FAs transportation are also relevant as cancer biomarkers. Carnitine palmitoyltransferase, CPT1A, is a protein that is responsible for the translocation of FAs from the cytosol to the mitochondrial matrix, where FA oxidation occurs. Associations of shorter disease-free survival with CPT1A positivity in invasive lobular carcinoma of the breast have been found (142).

Another study recognized a gene expression signature composed of 19 genes associated with FAO that was significantly associated with breast cancer patient survival. These 19 genes are referred to as the “fatty acid oxidation (FAO)” signature. Included in this signature were genes that have previously been identified as the core components of the FA β-oxidation pathway, such as *CPT1A*. Moreover, the expression of CPT1A was elevated in estrogen receptor–positive, compared to estrogen receptor–negative tumors and cell lines (143). Data from TCGA clearly confirm a *CPT1A* association with poor prognosis in breast cancer, whereas *CPT1A* is a marker of good prognosis in renal cancer and *CPT1C* in pancreas (Table 1).

Other relevant FA transporter is CD36. CD36, a scavenger receptor expressed in multiple cell types, mediates lipid uptake, immunological recognition, inflammation, molecular adhesion, and apoptosis. CD36 has been continually proposed as a prognostic marker in diverse cancers, mostly of epithelial origin (breast, prostate, ovary, and colon) and also for hepatic carcinoma and gliomas (141). Through systematic analysis of the multiple omics data from TCGA, it has been found that the most widely altered lipid metabolism pathways in pan-cancer are FA metabolism, AA metabolism, cholesterol metabolism, and peroxisome proliferator-activated receptor (PPAR) signaling. Genes related to lipid metabolism and immune response that were associated with poor prognosis were discovered including *CD36* (188).

### Cholesterol-Related Alterations as Biomarkers of Cancer Prognosis and Survival

First step of cholesterol or mevalonate pathway is catalyzed by acetyl-coenzyme A ACAT1 (Figure 2). ACAT1 is a mitochondrial enzyme that catalyzes the reversible formation of acetoacetyl-CoA from two molecules of AcCoA. An increased expression of ACAT1 in intratumor cholesteryl ester–rich breast tumors was reported (189). Also it has been proposed that ACAT1 expression could serve as a potential prognostic marker in prostate cancer, specifically in differentiating indolent and aggressive forms of cancer (144, 145). Data from TCGA suggest that *ACAT1* is a marker of good prognosis in liver and renal tumors. Interestingly, isoform 2 (*ACAT2*) is a marker of good prognosis in CRCs, whereas in endometrial and renal tumors, *ACAT2* has the opposite effect (Table 1).

Next step in cholesterol synthesis is mediated by 3-hydroxy-3-methylglutaryl-CoA synthase (HMGCS). This enzyme, with two isoforms, condenses AcCoA with acetoacetyl-CoA to form HMG-CoA, which is the substrate for HMG-CoA reductase. *HMGCS2* expression is associated with reduced clinical prognosis and outcomes in patients with CRC and oral squamous cell carcinoma. It has been suggested that HMGCS2 may act as a helpful prognostic marker and essential target for potential therapeutic strategies against advanced cancer (146). Also, it has been described that HMGCS2 works as a tumor suppressor and has a prognostic impact in prostate cancer, capable of predicting the risk of biochemical recurrence (147). However, in TCGA population, both isoforms are favorable makers of renal cancer. Besides, *HMGCS2* determines good prognosis in ovarian and liver cancer (Table 1).

HMGCR is the rate-limiting enzyme of the mevalonate pathway (Figure 2). HMG-CoA reductase expression in CRC and breast cancer correlates with favorable clinicopathological characteristics and an improved clinical outcome (148–150). Besides, *HMCGR* expression is a predictor of response to tamoxifen in breast cancer (190) and also may predict patient response to radiotherapy in ductal carcinoma *in situ* (191). In TCGA subset, *HMGCR* also is a good prognosis marker of renal tumors (Table 1). Statins, lipid-lowering compounds commonly used in cardiovascular disease, are competitive inhibitors of HMGCR. The value of HMGCR as a predictor of response to neoadjuvant or adjuvant statin treatment in cancer was also studied (192).

Once that cholesterol is synthesized, there are several cholesterol transporter proteins that play key roles in cholesterol and phospholipids homeostasis. The ATP-binding cassette transporter ABCA1 is a transmembrane protein responsible for the reverse cholesterol transport from the inner cell to circulatory system. *ABCA1* is significantly overexpressed in patients of all stages of CRC, and its overexpression gives proliferative advantages together with caveolin-1–dependent increased migratory and invasive capacities (151). Individuals with upregulation of *ABCA1* expression have an improved risk of CRC recurrence and OS independently of tumor stage (8). *ABCA1* also forms part of the metabolic-signature ColoLipidGene able to precisely stratify stage II CRC with 5-fold higher risk of relapse (7). Moreover, the presence of tumoral genetic variants located in *ABCA1* coding region seems to be associated with CRC risk of death (8). In other tumor types, ABCA1 expression was related to positive lymph nodes, but not significantly associated with tumor recurrence or breast cancer–specific survival (193).

Together with ABCA1, ATP-binding cassette G1 (ABCG1) also initiates and propagates cellular cholesterol efflux. Several genetic variants in *ABCG1* have been associated with survival of NSCLC patients (194). Moreover, *ABCG1* expression seems to be a favorable prognostic marker of renal cancer in data from TCGA (Table 1).

Other members of the family are the ATP-binding cassettes G4, G5, and G8. High ABCG4 expression has been associated with poor prognosis in NSCLC patients treated with cisplatin-based chemotherapy (152). ABCG5 positivity in tumor buds have been proposed as an indicator of poor prognosis in node-negative CRC patients (153), whereas in TCGA tumors, *ABCG5* seems to have a favorable effect in liver prognosis (Table 1).

While cellular cholesterol efflux is mainly performed via ABCA1, cholesterol uptake is principally executed via the LDLR. The prognostic value of LDLR expression was analyzed in CRC where authors found that the absence of LDLR predicts a shorter survival (154). In the same line, lower LDLR expression was an independent prognostic factor associated with longer survival in patients with small cell lung cancer (195). By contrast, TCGA data suggest that *LDLR* could be a bad prognostic marker of pancreatic, renal, and urothelial cancers (Table 1).

### Lipid-Related Transcription Factor Alterations as Biomarkers of Cancer Prognosis and Survival

Five are the main transcription factors that regulate the expression of mediators of lipid metabolism: SREBP1, SREBP2, PPARγ, NR1H3, and NR1H2. Sterol regulatory element-binding protein 1 (SREBP1) is a known transcription factor of lipogenic genes, which plays important roles in regulating *de novo* lipogenesis. *SREBP1* is overexpressed and strongly associated with worse clinical outcomes in breast cancer (155). Moreover, SREBP1 also seems to have an essential role in pancreatic cancer, regulating tumorigenesis and being associated with bad prognosis (196). However, data from TCGA propose *SREBP1* as a favorable prognostic marker in pancreatic and endometrial cancers (Table 1).

The combined expression of sterol regulatory element-binding protein 2 (*SREBP2*) together with *HMGCR, NR1H3*, and *NR1H2* genes was associated with poor CRC clinical outcome independent of lymph node metastasis, distant metastasis, and advanced stage (156). Besides, expression of SREBP-2 was elevated in advanced pathologic grade and metastatic prostate cancer and significantly associated with poor clinical outcomes (157).

The PPARγ is a nuclear receptor that controls expression of mediators of lipid metabolism but also the inflammatory response. Additionally, it has been demonstrated that PPAR b/d and a isotypes also have important roles in FAO, FA storage, and cholesterogenesis (197).

Decreased expression of PPARγ has been observed in many tumor types. In this sense, reduced PPARγ expression within the tumor is associated with poor prognosis in lung cancer patients (158, 159). In the same line, tumor expression of PPARγ is independently associated with increased survival of CRC patients (160). Also in patients with breast and prostate cancer, PPARγ is a marker of better prognosis and is associated with better survival (161–163). Importantly, one study reports that cytoplasmic PPARγ expression appeared as an independent marker of poor prognosis in primary breast cancers (198). TCGA analysis proposed PPARγ as a favorable prognostic marker for renal and urothelial cancers (Table 1).

Finally, several studies have also evaluated the association between PPARγ genetic variants and the risk of CRC (199). In patients with stages II/III CRC, polymorphism rs1801282 in PPARγ was significantly associated with tumor recurrence (200).

*NR1H3* and *NR1H2* encode for liver X receptor (LXR) α and LXR β, respectively. They are intimately related nuclear receptors that react to elevated levels of intracellular cholesterol by enhancing transcription of genes that control cholesterol efflux and FA biosynthesis. *NR1H3* expression was significantly correlated to better survival in completely resected stages II and III NSCLC patients (164). Moreover, one study reports that *NR1H3* and *NR1H2* belong to a transcription signature associated with poor CRC clinical outcome independent of lymph node metastasis, distant metastasis, and advanced stage (156). This result is validated in TCGA dataset (Table 1) where *NR1H2* was also associated with CRC poor prognosis.

## Targeting the Altered Lipid Metabolism in Cancer

Because of the essential role of FAs for cancer cell proliferation and progression, drugs to target lipogenic enzymes and/or transcription factors regulating the intracellular lipid homeostasis are considering as promising therapeutic strategies against cancer.

Different drugs have been already evaluated to target (i) lipogenic enzymes (FASN, ACLY, ACC); (ii) the exogenous lipid uptake (LXR, CD36, FABP4/5); (iii) inflammatory signaling pathways (PTGS2); (iv) regulation of intracellular lipid homeostasis (PPARγ, CPT1a, lipin2, HSL, MAGAT, DAGAT…); and/or (v) saturated vs. unsaturated FAs. Their efficacy has been demonstrated in numerous models of cancer, including *in vitro* preclinical and clinical studies.

In Table 2, we summarize main drugs evaluated in preclinical and clinical studies. Nevertheless, although the results of these studies are encouraging, side effects due to the many different regulatory mechanisms of lipid metabolism are still a big challenge.

**Table 2:** Preclinical and clinical studies with main drugs evaluated to target the altered lipid metabolism in cancer.

**Target**	**Drug**	**Type of cancer**	**Preclinical/clinical trial**	
FASN	Cerulenin	Breast Cancer		(48)
		Ovarian Cancer		(201)
	C75	Renal Cancer		(59)
		Breast Cancer		(53)
		Lung Cancer		(43)
	Orlistat	Melanoma		(57, 202)
		Prostate Cancer		(86)
	Fasnall	Breast Cancer		(87)
	C93	NSCLC		(42, 43)
	C247	Breast Cancer		(44)
	TV3166	CRC		(45)
	TVB-2640	NSCLC	NCT03808558	(56)
		TNBC	NCT03179904	(56)
		HG Astrocytoma	NCT03032484	(203)
		Ovarian, Breast Cancer	NCT02223247	(204)
	Triclosan	Breast		(58, 60)
ACLY	SB-204990	NSCLC, Prostate, Ovarian		(51)
		NSCLC		(61)
ACC1/2	ND-630 (GS-0976)	NASH		(71)
	TOFA	HNSCC		(205)
		Ovarian		(33)
	ND-654	HCC		(34)
	GS-0976	NASH		(36)
			NCT02856555	(35)
	ND-646	NSCL		(206)
SCD1	CVT-12	HCC		(207)
	SSI-4	HCC		(208)
	Betulinic acid	CRC		(209)
		GBC		(210)
	MF-438	NSCLC		(211)
	A939572	NSCLC		(212)
		ccRCC		(213)
		Prostate		(213)
CPT1A	Etomoxir	Leukemia		(214)
	Ranolazine	Prostate Cancer		(215)
		Glioblastoma		(216)
	Etomoxir, Ranolazine, Perhexiline	Prostate Cancer		(217)
	Perhexiline	CLL		(218)
		Breast Cancer		(219, 220)
SREBP	Betulin	HCC		(221)
		Melanoma		(222)
	Fatostatin	Prostate		(223, 224)
		Glioma		(225)
		HCC		(226)
LXR	T0901317/GW3965	BPDCN		(227)
	LXR623 and GW3965	Colon/Glioblastoma		(228)
	GW3965	Glioma		(229)
ACAT1	Avasimive	Prostate/Colon Cancer		(230)
		GBM		(231)
		CML		(232)
CD36	FA6.152	Oral Cancer		(80)
		Prostate Cancer		(233)
HMGCR	Fluvastatin	Prostate	NCT01992042	(234)
			NCT00608595	
	Simvastatin	CRC	NCT00994903	(235)
		NSCLC	NCT00452244	(236)
MAGL	URB602	Colon		(237)
PTGS2	Celecoxib	Lung Cancer		(238)
		Ovarian Cancer (HFD)		(239)
		NSCLC	NCT00046839	(+)
		PDAC	NCT01111591	(240)
		Prostate cancer	NCT00073970	(+)
		Early CRC	NCT00608595	(+)
PPARG	VSP-17	Breast Cancer		(241)
FABP4	BMS309403	HCC		(242)
		Prostate Cancer		(243)
	FABP5	SBFI26	CRPC	(244)

*(+) Unpublished results*.

Recently, there is growing interest on complementary approaches by means of dietary interventions for cancer treatment. The success of such interventions requires a deep knowledge of the metabolic requirements of tumors, considering the nutritional status of the individuals—obesity, metabolic syndrome and/or insulin resistance, among others—and the genetic susceptibilities to metabolic alterations. Moreover, the knowledge of the molecular targets and mechanism of action of dietary ingredients will be crucial to apply these approaches with the conventional chemotherapy in order to improve the responses to the clinical treatments and the well-being of patients.

Precision nutrition should be considered at three levels: (1) nutritional guidelines based on age, gender, and other sociocultural factors; (2) individualized recommendations after refined phenotyping; and a (3) genetic-nutrition based on genetic variants with high penetrance and on the response to nutritional interventions (6).

The improvement of the “omics” sciences, including transcriptomics, proteomics, metabolomics, lipidomics, and metagenomics, provides a more complete scenario for personalized nutritional interventions (13, 245). The main challenge is to define tumor heterogeneities, which can be originated by genomic, epigenomic, transcriptomic, and immune variability. This will lead to patients' stratification for personalized treatments in the clinics (246).

Nutrigenetics aims to study the effect of genetic variants on the dietary response and the risk of several diseases. For example, SNPs in the *CD36* gene associate with dyslipidemia when high amounts of fats are consumed (247). In addition, dietary ingredients affect cancer risk and progression affecting gene expression. Nutrigenomics considers the effect of diet-derived ingredients on gene expression and, consequently, on the proteome and metabolome.

Dietary ingredients and nutrients from natural sources, such as epigallocatechin-3-gallate, curcumin, sulforaphane, and genistein, have been shown to have anticancer properties regulating the expression of genes related to cancer. Polyphenols contribute to the prevention of obesity through the modulation of genes implicated in adipogenesis, lipolysis, and FAO (248–251).

Importantly, in the frame of precision nutrition, dietary interventions might also provide systemic responses affecting the antitumoral response of the immune system, as well as the reduction of low-grade chronic inflammation, dyslipidemia, insulin resistance, and/or obesity.

The direct association of diet with obesity and dysbiosis requires further research to understand the impact of diet on cancer prognosis. High intake of saturated FAs increases the expression of genes related to inflammation, insulin resistance, and/or hepatic steatosis. In contrast, Mediterranean diet downregulates the expression of genes related to oxidative stress, inflammation, and/or insulin signaling (252, 253). Importantly, high levels of triglycerides and LDLs have been associated with CRC prognosis and distant metastasis. Cholesterol in high-fat diets associates with colorectal tumorigenesis (254). Ceramide sphingolipids have been shown to be antitumoral in combination with tamoxifen (255). Phosphatidylcholine is increased in CRC cells. Increased intake of MUFAs is associated with reduce inflammation in CRC cancer (256). Energy-restricted diets supplemented with EPA and α-lipoic acid increase the expression of FAO genes, diminishing the expression of genes related to *de novo* lipogenesis and inflammation (257) (Table 3).

**Table 3:** Preclinical and clinical studies with bioactive compounds from natural sources to target the altered lipid metabolism and/or associated risk factors (mainly obesity and T2DM) in cancer.

**Family**	**Bioactive compounds**	**Molecular targets, metabolic effects**	**Preclinical/clinical trials**	**References**
**Polyphenols**			
Flavonoids	Gallic acid and its derivatives EGCG, gallate, ethyl gallate, gallocatechin gallate, methyl gallate, propyl gallate, theaflavin-3-gallate	↑AMPK, FAO, thermogenesis		(258)
		↓antiobesity		(259)
		↓Cholesterol, LDL	NCT02147041	(260)
		↓lipogenesis, ↓PPARG, LXR, ↑AMPK		(261, 262)
		↑AMPK, SIRT, PGC1a, FAO, UCP1, CYp7a1		(263)
		↓dyslipidemia		(264)
		↓dyslipidemia	NCT02627898	(265)
		↑FAO, ↓antiobesity	NCT02381145	(266)
		↓HOMAIR, T2DM	Human study	(267)
	**Citrus flavonoids**			
	Nobilettin	↓HSL, ACC, ↑AMPK, CPT1a, ACOX1, FAO		(268)
	Naringenin	↑PPARα, CPT-1, UCP-2, FAO, ↓SREBP1c, 3HMGCR, hepatic steatosis		(269–272)
	Tangeretin	↑PPARα, FAO		(273)
	Hesperetin	↑PPARα, PPARγ, AMPK, FAO, ↓lipogenesis		(274)
	Baicalin	↓SREBP-1c, FASN, ACC		(275)
	Hispidulin	↑PPARα, CPT1α ↑Acat1, Acad1, HMGCS2		(276, 277)
	Mangiferin	↓inflammation, T2DM, steatosis, ACC, DGAT2, ↑ FAO(CPT1a)	
	Dihydromyricetin	↓hepatic steatosis	ChiCTRTRC12002377	(278)
	Berberin	↓hepatic steatosis, TG and cholesterol levels	NCT00633282	(279)
	Luteolin	↑FAO, ↓lipogenesis, cholesterogenesis, HMGCS1	NCT00633282	(280)
	Quercetin	↓ CYP2E1, inflammation, obesity, T2DM		(281, 282)
Stilbenos	Resveratrol	↓ steatosis, adipogenesis, SREBP1c, lipin1, ACC, ↑AMPK, SIRT1, FAO	(283–285)
Curcuminoids	Curcumin	↓steatosis, adipogenesis, SREBP1c, FASN, SCD1, GPAT-1, ↑1AMPK, FAO	(286, 287)
Phenolic acids	Ellagic acid	↓steatosis, Insulin resistance		(288)
				
**Terpenoids**			
	Carnosol	↓hyperglycemia, inflammation, lipogenesis, anticancer		(289, 290)
	Betulinic acid	↓SCD, steatosis, lipogenesis		(209)
	Ursolic acid	↑AMPK, FAO, ↓lipogenesis		(291)
	Ginsenoside	↑AMPK, perilipin, FAO		(292–294)
	Licopene	↓inflammation	ISRCTN99660610	(295)

Importantly, the efficacy of fasting cycles or cycles of fasting mimicking diets in dampening tumor development has already been established (296), and the implementation of other dietary approaches for cancer therapy is likely to take a similar approach.

## Concluding Remarks

Metabolic alterations of tumors have been well-recognized as one of the hallmarks of cancer. At present, several investigations have demonstrated the consequences of lipid metabolism deregulation in cancer not only sustain tumor growth but also promote cell migration, invasion, and angiogenesis. In this review, we have discussed about the main lipid metabolism alterations found in cancer by describing their mechanism of action and their oncologic implications. Importantly, we emphasize the crucial role of the aberrant lipid metabolism not only affecting the primary tumors but also shaping the tumor microenvironment to promote malignancy and dissemination. Moreover, we have explored the available public data bases containing mRNA data (TCGA) and protein expression data (The Human Protein Atlas) to obtain a global view of the putative implications of lipid metabolism–related genes in cancer prognosis of the most frequent types of cancer according to the WHO: lung, CRC, breast, and prostate cancers.

We also highlight the relevance of “omics” technologies, including genomic and transcriptomic data, considering the phenotypic metabolic status (mainly obesity) to define lipid metabolic scores to be integrated into the clinical advice. Thus, the use of this knowledge will allow a better stratification of patients, which will be translated into improvements on the OS and well-being of the patients. In the frame of precision medicine, new clinical trials integrating classical chemotherapies with precision nutrition–based strategies—bioactive products and diet derived nutrients—will provide an unquestionable line of research in cancer treatment.

## Author Contributions

LPF and MGC wrote the paper. AR performed the critical revision of the article. All authors conceptually designed the manuscript.

## Conflict of Interest

The authors declare that the research was conducted in the absence of any commercial or financial relationships that could be construed as a potential conflict of interest.

## References

[1] Siegel RL Miller KD Jemal A Cancer statistics, 2020. Cancer J Clin. (2020) 70:7–30. 10.3322/caac.21590 31912902

[2] Hanahan D Weinberg RA. Hallmarks of cancer: the next generation. Cell. (2011) 144:646–74. 10.1016/j.cell.2011.02.013 21376230

[3] Beloribi-Djefaflia S Vasseur S Guillaumond F. Lipid metabolic reprogramming in cancer cells. Oncogenesis. (2016) 5:e189. 10.1038/oncsis.2015.49 26807644 PMC4728678

[4] van Meer G Voelker DR Feigenson GW. Membrane lipids: where they are and how they behave. Nat Rev Mol Cell Biol. (2008) 9:112–24. 10.1038/nrm2330 18216768 PMC2642958

[5] Peck B Schulze A. Lipid metabolism at the nexus of diet and tumor microenvironment. Trends in Cancer. (2019) 5:693–703. 10.1016/j.trecan.2019.09.007 31735288

[6] Gómez de Cedrón M Ramírez de Molina A Chapter 28 - Precision nutrition to target lipid metabolism alterations in cancer. In: Faintuch J, Faintuch S. editors. Precision Medicine for Investigators, Practitioners Providers. Academic Press (2019). p. 291–9. 10.1016/B978-0-12-819178-1.00028-9

[7] Vargas T Moreno-Rubio J Herranz J Cejas P Molina S González-Vallinas M. ColoLipidGene: signature of lipid metabolism-related genes to predict prognosis in stage-II colon cancer patients. Oncotarget. (2015) 6:7348–63. 10.18632/oncotarget.3130 25749516 PMC4466690

[8] Fernández LP Ramos-Ruiz R Herranz J Martín-Hernández R Vargas T Mendiola M. The transcriptional and mutational landscapes of lipid metabolism-related genes in colon cancer. Oncotarget. (2017) 9:5919–30. 10.18632/oncotarget.23592 29464044 PMC5814184

[9] Sánchez-Martínez R Cruz-Gil S Gómez de Cedrón M Álvarez-Fernández M Vargas T Molina S. A link between lipid metabolism and epithelial-mesenchymal transition provides a target for colon cancer therapy. Oncotarget. (2015) 6:38719–36. 10.18632/oncotarget.5340 26451612 PMC4770732

[10] Fisher KE Pop A Koh W Anthis NJ Saunders WB Davis GE. Tumor cell invasion of collagen matrices requires coordinate lipid agonist-induced G-protein and membrane-type matrix metalloproteinase-1-dependent signaling. Mol Cancer. (2006) 5:69. 10.1186/1476-4598-5-69 17156449 PMC1762019

[11] Luo X Cheng C Tan Z Li N Tang M Yang L. Emerging roles of lipid metabolism in cancer metastasis. Mol Cancer. (2017) 16:76. 10.1186/s12943-017-0646-3 28399876 PMC5387196

[12] Rung J Brazma A. Reuse of public genome-wide gene expression data. Nat Rev Genet. (2013) 14:89–99. 10.1038/nrg3394 23269463

[13] Ferguson LR De Caterina R Görman U Allayee H Kohlmeier M Prasad C. Guide and position of the international society of nutrigenetics/nutrigenomics on personalised nutrition: part 1 - fields of precision nutrition. J Nutrigenet Nutrigenomics. (2016) 9:12–27. 10.1159/000445350 27169401

[14] Aguirre-Portolés C Fernández LP Ramírez de Molina A. precision nutrition for targeting lipid metabolism in colorectal cancer. Nutrients. (2017) 9:1076. 10.3390/nu9101076 28956850 PMC5691693

[15] Simonds NI Ghazarian AA Pimentel CB Schully SD Ellison GL Gillanders EM. Review of the gene-environment interaction literature in cancer: what do we know?. Genet Epidemiol. (2016) 40:356–365. 10.1002/gepi.21967 27061572 PMC4911236

[16] Gonzalez-Muniesa P Martinez-Gonzalez MA Hu FB Despres JP Matsuzawa Y Loos RJF Obesity. Nat Rev Dis Primers. (2017) 3:17034 10.1038/nrdp.2017.34 28617414

[17] Martin-Timon I Sevillano-Collantes C Segura-Galindo A Del Canizo-Gomez FJ. Type 2 diabetes and cardiovascular disease: have all risk factors the same strength? World J Diabetes. (2014) 5:444–70. 10.4239/wjd.v5.i4.444 25126392 PMC4127581

[18] Shalitin S Battelino T Moreno LA. Obesity, metabolic syndrome and nutrition. World Rev Nutr Diet. (2016) 114:21–49. 10.1159/000441810 26906026

[19] Calle EE Rodriguez C Walker-Thurmond K Thun MJ. Overweight, obesity, and mortality from cancer in a prospectively studied cohort of U.S. adults. N Engl J Med. (2003) 348:1625–38. 10.1056/NEJMoa021423 12711737

[20] Zhang X Zhou G Sun B Zhao G Liu D Sun J. Impact of obesity upon prostate cancer-associated mortality: a meta-analysis of 17 cohort studies. Oncol Lett. (2015) 9:1307–12. 10.3892/ol.2014.2841 25663903 PMC4315023

[21] Zhang Y Liu H Yang S Zhang J Qian L Chen X. Overweight, obesity and endometrial cancer risk: results from a systematic review and meta-analysis. Int J Biol Markers. (2014) 29:e21–9. 10.5301/jbm.5000047 24170556

[22] Dobbins M Decorby K Choi BC. The association between obesity and cancer risk: a meta-analysis of observational studies from 1985 to 2011. ISRN Prev Med. (2013) 2013:680536. 10.5402/2013/680536 24977095 PMC4062857

[23] Liu Z Zhang TT Zhao JJ Qi SF Du P Liu DW. The association between overweight, obesity and ovarian cancer: a meta-analysis. Jpn J Clin Oncol. (2015) 45:1107–15. 10.1093/jjco/hyv150 26491203

[24] Qin Q Xu X Wang X Zheng XY. Obesity and risk of bladder cancer: a meta-analysis of cohort studies. Asian Pac J Cancer Prev. (2013) 14:3117–21. 10.7314/apjcp.2013.14.5.3117 23803089

[25] Larsson SC Wolk A. Overweight, obesity and risk of liver cancer: a meta-analysis of cohort studies. Br J Cancer. (2007) 97:1005–8. 10.1038/sj.bjc.6603932 17700568 PMC2360408

[26] Moghaddam AA Woodward M Huxley R. Obesity and risk of colorectal cancer: a meta-analysis of 31 studies with 70,000 events. Cancer Epidemiol Biomarkers Prev. (2007) 16:2533–47. 10.1158/1055-9965.EPI-07-0708 18086756

[27] Ritchie SA Connell JM. The link between abdominal obesity, metabolic syndrome and cardiovascular disease. Nutr Metab Cardiovasc Dis. (2007) 17:319–26. 10.1016/j.numecd.2006.07.005 17110092

[28] Schapira DV Clark RA Wolff PA Jarrett AR Kumar NB Aziz NM. Visceral obesity and breast cancer risk. Cancer. (1994) 74:632–9. 10.1002/1097-0142(19940715)74:2<632::aid-cncr2820740215>3.0.co;2-t 8033042

[29] Nieman KM Kenny HA Penicka CV Ladanyi A Buell-Gutbrod R Zillhardt MR. Adipocytes promote ovarian cancer metastasis and provide energy for rapid tumor growth. Nat Med. (2011) 17:1498–503. 10.1038/nm.2492 22037646 PMC4157349

[30] Hanahan D Coussens LM. Accessories to the crime: functions of cells recruited to the tumor microenvironment. Cancer Cell. (2012) 21:309–22. 10.1016/j.ccr.2012.02.022 22439926

[31] Martinez-Outschoorn UE Pestell RG Howell A Tykocinski ML Nagajyothi F Machado FS Energy transfer in “parasitic” cancer metabolism: mitochondria are the powerhouse and achilles' heel of tumor cells. Cell Cycle. (2011) 10:4208–16. 10.4161/cc.10.24.18487 22033146 PMC3272257

[32] Park J Cho SY Lee SB Son H Jeong H Obesity is associated with higher risk of prostate cancer detection in a biopsy population in Korea. BJU Int. (2014) 114:891–5. 10.1111/bju.12600 24314095

[33] Li S Qiu L Wu B Shen H Zhu J Zhou L. TOFA suppresses ovarian cancer cell growth *in vitro* and *in vivo*. Mol Med Rep. (2013) 8:373–8. 10.3892/mmr.2013.1505 23732836

[34] Lally JSV Ghoshal S DePeralta DK Moaven O Wei L Masia R. Inhibition of acetyl-CoA carboxylase by phosphorylation or the inhibitor ND-654 suppresses lipogenesis and hepatocellular carcinoma. Cell Metab. (2019) 29:174–82.e5. 10.1016/j.cmet.2018.08.020 30244972 PMC6643297

[35] Loomba R Kayali Z Noureddin M Ruane P Lawitz EJ Bennett M. GS-0976 reduces hepatic steatosis and fibrosis markers in patients with nonalcoholic fatty liver disease. Gastroenterology. (2018) 155:1463–73.e6. 10.1053/j.gastro.2018.07.027 30059671 PMC6318218

[36] Lu T Sun L Wang Z Zhang Y He Z Xu C. Fatty acid synthase enhances colorectal cancer cell proliferation and metastasis via regulating AMPK/mTOR pathway. Onco Targets Ther. (2019) 12:3339–47. 10.2147/OTT.S199369 31118685 PMC6504633

[37] Louis P Hold GL Flint HJ. The gut microbiota, bacterial metabolites and colorectal cancer. Nat Rev Microbiol. (2014) 12:661–72. 10.1038/nrmicro3344 25198138

[38] Sheflin AM Whitney AK Weir TL. Cancer-promoting effects of microbial dysbiosis. Curr Oncol Rep. (2014) 16:406. 10.1007/s11912-014-0406-0 25123079 PMC4180221

[39] Chen JQ Russo J. Dysregulation of glucose transport, glycolysis, TCA cycle and glutaminolysis by oncogenes and tumor suppressors in cancer cells. Biochim Biophys Acta. (2012) 1826:370–84. 10.1016/j.bbcan.2012.06.004 22750268

[40] Warburg O. On the origin of cancer cells. Science. (1956) 123:309–14. 10.1126/science.123.3191.309 13298683

[41] DeBerardinis RJ Lum JJ Hatzivassiliou G Thompson CB. The biology of cancer: metabolic reprogramming fuels cell growth and proliferation. Cell Metab. (2008) 7:11–20. 10.1016/j.cmet.2007.10.002 18177721

[42] Orita H Coulter J Lemmon C Tully E Vadlamudi A Medghalchi SM. Selective inhibition of fatty acid synthase for lung cancer treatment. Clin Cancer Res. (2007) 13:7139–45. 10.1158/1078-0432.CCR-07-1186 18056164

[43] Orita H Coulter J Tully E Kuhajda FP Gabrielson E. Inhibiting fatty acid synthase for chemoprevention of chemically induced lung tumors. Clin Cancer Res. (2008) 14:2458–64. 10.1158/1078-0432.CCR-07-4177 18413838

[44] Alli PM Pinn ML Jaffee EM McFadden JM Kuhajda FP. Fatty acid synthase inhibitors are chemopreventive for mammary cancer in neu-N transgenic mice. Oncogene. (2005) 24:39–46. 10.1038/sj.onc.1208174 15489885

[45] Zaytseva YY Rychahou PG Le AT Scott TL Flight RM Kim JT. Preclinical evaluation of novel fatty acid synthase inhibitors in primary colorectal cancer cells and a patient-derived xenograft model of colorectal cancer. Oncotarget. (2018) 9:24787–800. 10.18632/oncotarget.25361 29872506 PMC5973868

[46] Mashima T Sato S Okabe S Miyata S Matsuura M Sugimoto Y. Acyl-CoA synthetase as a cancer survival factor: its inhibition enhances the efficacy of etoposide. Cancer Sci. (2009) 100:1556–62. 10.1111/j.1349-7006.2009.01203.x 19459852 PMC11158289

[47] Menendez JA Vellon L Espinoza I Lupu R. The metastasis inducer CCN1 (CYR61) activates the fatty acid synthase (FASN)-driven lipogenic phenotype in breast cancer cells. Oncoscience. (2016) 3:242–57. 10.18632/oncoscience.314 27713913 PMC5043073

[48] Pizer ES Jackisch C Wood FD Pasternack GR Davidson NE Kuhajda FP. Inhibition of fatty acid synthesis induces programmed cell death in human breast cancer cells. Cancer Res. (1996) 56:2745–7 8665507

[49] Roongta UV Pabalan JG Wang X Ryseck RP Fargnoli J Henley BJ. Cancer cell dependence on unsaturated fatty acids implicates stearoyl-CoA desaturase as a target for cancer therapy. Mol Cancer Res. (2011) 9:1551–61. 10.1158/1541-7786.MCR-11-0126 21954435

[50] Santos CR Schulze A. Lipid metabolism in cancer. FEBS J. (2012) 279:2610–3. 10.1111/j.1742-4658.2012.08644.x 22621751

[51] Hatzivassiliou G Zhao F Bauer DE Andreadis C Shaw AN Dhanak D. ATP citrate lyase inhibition can suppress tumor cell growth. Cancer Cell. (2005) 8:311–21. 10.1016/j.ccr.2005.09.008 16226706

[52] Menendez JA Lupu R. Fatty acid synthase and the lipogenic phenotype in cancer pathogenesis. Nat Rev Cancer. (2007) 7:763–77. 10.1038/nrc2222 17882277

[53] Menendez JA Vellon L Colomer R Lupu R. Pharmacological and small interference RNA-mediated inhibition of breast cancer-associated fatty acid synthase (oncogenic antigen-519) synergistically enhances taxol (paclitaxel)-induced cytotoxicity. Int J Cancer. (2005) 115:19–35. 10.1002/ijc.20754 15657900

[54] Rao S Lowe M Herliczek TW Keyomarsi K. Lovastatin mediated G1 arrest in normal and tumor breast cells is through inhibition of CDK2 activity and redistribution of p21 and p27, independent of p53. Oncogene. (1998) 17:2393–402. 10.1038/sj.onc.1202322 9811471

[55] Clendening JW Pandyra A Boutros PC El Ghamrasni S Khosravi F Trentin GA. Dysregulation of the mevalonate pathway promotes transformation. Proc Natl Acad Sci USA. (2010) 107:15051–6. 10.1073/pnas.0910258107 20696928 PMC2930553

[56] Che L Paliogiannis P Cigliano A Pilo MG Chen X Calvisi DF. Pathogenetic, prognostic, and therapeutic role of fatty acid synthase in human hepatocellular carcinoma. Front Oncol. (2019) 9:1412. 10.3389/fonc.2019.01412 31921669 PMC6927283

[57] Seguin F Carvalho MA Bastos DC Agostini M Zecchin KG Alvarez-Flores MP. The fatty acid synthase inhibitor orlistat reduces experimental metastases and angiogenesis in B16-F10 melanomas. Br J Cancer. (2012) 107:977–87. 10.1038/bjc.2012.355 22892389 PMC3464771

[58] Sadowski MC Pouwer RH Gunter JH Lubik AA Quinn RJ Nelson CC. The fatty acid synthase inhibitor triclosan: repurposing an anti-microbial agent for targeting prostate cancer. Oncotarget. (2014) 5:9362–81. 10.18632/oncotarget.2433 25313139 PMC4253440

[59] Horiguchi A Asano T Ito K Sumitomo M Hayakawa M. Pharmacological inhibitor of fatty acid synthase suppresses growth and invasiveness of renal cancer cells. J Urol. (2008) 180:729–36. 10.1016/j.juro.2008.03.186 18555493

[60] Lee HR Hwang KA Nam KH Kim HC Choi KC. Progression of breast cancer cells was enhanced by endocrine-disrupting chemicals, triclosan and octylphenol, via an estrogen receptor-dependent signaling pathway in cellular and mouse xenograft models. Chem Res Toxicol. (2014) 27:834–42. 10.1021/tx5000156 24684733

[61] Migita T Narita T Nomura K Miyagi E Inazuka F Matsuura M. ATP citrate lyase: activation and therapeutic implications in non-small cell lung cancer. Cancer Res. (2008) 68:8547–54. 10.1158/0008-5472.CAN-08-1235 18922930

[62] Carracedo A Cantley LC Pandolfi PP. Cancer metabolism: fatty acid oxidation in the limelight. Nat Rev Cancer. (2013) 13:227–32. 10.1038/nrc3483 23446547 PMC3766957

[63] Khasawneh J Schulz MD Walch A Rozman J Hrabe de Angelis M Klingenspor M. Inflammation and mitochondrial fatty acid beta-oxidation link obesity to early tumor promotion. Proc Natl Acad Sci USA. (2009) 106:3354–9. 10.1073/pnas.0802864106 19208810 PMC2651311

[64] Freigang S Ampenberger F Weiss A Kanneganti TD Iwakura Y Hersberger M. Fatty acid-induced mitochondrial uncoupling elicits inflammasome-independent IL-1alpha and sterile vascular inflammation in atherosclerosis. Nat Immunol. (2013) 14:1045–53. 10.1038/ni.2704 23995233

[65] Li S Zhou T Li C Dai Z Che D Yao Y. High metastaticgastric and breast cancer cells consume oleic acid in an AMPK dependent manner. PLoS ONE. (2014) 9:e97330. 10.1371/journal.pone.0097330 24823908 PMC4019637

[66] Ludtmann MH Angelova PR Zhang Y Abramov AY Dinkova-Kostova AT. Nrf2 affects the efficiency of mitochondrial fatty acid oxidation. Biochem J. (2014) 457:415–24. 10.1042/BJ20130863 24206218 PMC4208297

[67] Pike LS Smift AL Croteau NJ Ferrick DA Wu M. Inhibition of fatty acid oxidation by etomoxir impairs NADPH production and increases reactive oxygen species resulting in ATP depletion and cell death in human glioblastoma cells. Biochim Biophys Acta. (2011) 1807:726–34. 10.1016/j.bbabio.2010.10.022 21692241

[68] Carracedo A Weiss D Leliaert AK Bhasin M de Boer VCJ Laurent G. A metabolic prosurvival role for PML in breast cancer. J Clin Invest. (2012) 122:3088–100. 10.1172/JCI62129 22886304 PMC3433768

[69] Przybytkowski E Joly E Nolan CJ Hardy S Francoeur AM Langelier Y. Upregulation of cellular triacylglycerol - free fatty acid cycling by oleate is associated with long-term serum-free survival of human breast cancer cells. Biochem Cell Biol. (2007) 85:301–10. 10.1139/o07-001 17612624

[70] Geng F Guo D. Lipid droplets, potential biomarker and metabolic target in glioblastoma. Intern Med Rev. (2017) 3. 10.18103/imr.v3i5.443 29034362 PMC5639724

[71] Harriman G Greenwood J Bhat S Huang X Wang R Paul D. Acetyl-CoA carboxylase inhibition by ND-630 reduces hepatic steatosis, improves insulin sensitivity, and modulates dyslipidemia in rats. Proc Natl Acad Sci USA. (2016) 113:E1796–805. 10.1073/pnas.1520686113 26976583 PMC4822632

[72] Togashi A Katagiri T Ashida S Fujioka T Maruyama O Wakumoto Y. Hypoxia-inducible protein 2 (HIG2), a novel diagnostic marker for renal cell carcinoma and potential target for molecular therapy. Cancer Res. (2005) 65:4817–26. 10.1158/0008-5472.CAN-05-0120 15930302

[73] Penrose H Heller S Cable C Makboul R Chadalawada G Chen Y. Epidermal growth factor receptor mediated proliferation depends on increased lipid droplet density regulated via a negative regulatory loop with FOXO3/Sirtuin6. Biochem Biophys Res Commun. (2016) 469:370–6. 10.1016/j.bbrc.2015.11.119 26657850 PMC5607009

[74] Nomura DK Long JZ Niessen S Hoover HS Ng SW Cravatt BF. Monoacylglycerol lipase regulates a fatty acid network that promotes cancer pathogenesis. Cell. (2010) 140:49–61. 10.1016/j.cell.2009.11.027 20079333 PMC2885975

[75] Ou J Miao H Ma Y Guo F Deng J Wei X. Loss of abhd5 promotes colorectal tumor development and progression by inducing aerobic glycolysis and epithelial-mesenchymal transition. Cell Rep. (2018) 24:2795–97. 10.1016/j.celrep.2018.08.050 30184511 PMC6186214

[76] Nomura DK Lombardi DP Chang JW Niessen S Ward AM Long JZ. Monoacylglycerol lipase exerts dual control over endocannabinoid and fatty acid pathways to support prostate cancer. Chem Biol. (2011) 18:846–56. 10.1016/j.chembiol.2011.05.009 21802006 PMC3149849

[77] Cheng X Geng F Pan M Wu X Zhong Y Wang C. Targeting DGAT1 ameliorates glioblastoma by increasing fat catabolism and oxidative stress. Cell Metabolism. (2020) 32:229–42.e8. 10.1016/j.cmet.2020.06.002 32559414 PMC7415721

[78] Amir M Czaja MJ. Autophagy in nonalcoholic steatohepatitis. Expert Rev Gastroenterol Hepatol. (2011) 5:159–66. 10.1586/egh.11.4 21476911 PMC3104297

[79] Kuemmerle NB Rysman E Lombardo PS Flanagan AJ Lipe BC Wells WA. Lipoprotein lipase links dietary fat to solid tumor cell proliferation. Mol Cancer Ther. (2011) 10:427–36. 10.1158/1535-7163.MCT-10-0802 21282354 PMC3074101

[80] Pascual G Avgustinova A Mejetta S Martín M Castellanos A Attolini CS-O. Targeting metastasis-initiating cells through the fatty acid receptor CD36. Nature. (2017) 541:41–5. 10.1038/nature20791 27974793

[81] Sundelin JP Stahlman M Lundqvist A Levin M Parini P Johansson ME. Increased expression of the very low-density lipoprotein receptor mediates lipid accumulation in clear-cell renal cell carcinoma. PLoS ONE. (2012) 7:e48694. 10.1371/journal.pone.0048694 23185271 PMC3501495

[82] Koizume S Miyagi Y. Lipid droplets: a key cellular organelle associated with cancer cell survival under normoxia and hypoxia. Int J Mol Sci. (2016) 17:1430. 10.3390/ijms17091430 27589734 PMC5037709

[83] Levi L Wang Z Doud MK Hazen SL Noy N. Saturated fatty acids regulate retinoic acid signalling and suppress tumorigenesis by targeting fatty acid-binding protein 5. Nat Commun. (2015) 6:8794. 10.1038/ncomms9794 26592976 PMC4662070

[84] Yue S Li J Lee SY Lee HJ Shao T Song B. Cholesteryl ester accumulation induced by PTEN loss and PI3K/AKT activation underlies human prostate cancer aggressiveness. Cell Metab. (2014) 19:393–406. 10.1016/j.cmet.2014.01.019 24606897 PMC3969850

[85] Rysman E Brusselmans K Scheys K Timmermans L Derua R Munck S. *De novo* lipogenesis protects cancer cells from free radicals and chemotherapeutics by promoting membrane lipid saturation. Cancer Res. (2010) 70:8117–26. 10.1158/0008-5472.CAN-09-3871 20876798

[86] Kridel SJ Axelrod F Rozenkrantz N Smith JW. Orlistat is a novel inhibitor of fatty acid synthase with antitumor activity. Cancer Res. (2004) 64:2070–5. 10.1158/0008-5472.can-03-3645 15026345

[87] Alwarawrah Y Hughes P Loiselle D Carlson DA Darr DB Jordan JL. Fasnall, a selective FASN inhibitor, shows potent anti-tumor activity in the MMTV-Neu model of HER2(+) Breast cancer. Cell Chem Biol. (2016) 23:678–88. 10.1016/j.chembiol.2016.04.011 27265747 PMC6443244

[88] Schug ZT Gottlieb E. Cardiolipin acts as a mitochondrial signalling platform to launch apoptosis. Biochim Biophys Acta. (2009) 1788:2022–31. 10.1016/j.bbamem.2009.05.004 19450542

[89] Zhao W Prijic S Urban BC Tisza MJ Zuo Y Li L. Candidate antimetastasis drugs suppress the metastatic capacity of breast cancer cells by reducing membrane fluidity. Cancer Res. (2016) 76:2037–49. 10.1158/0008-5472.CAN-15-1970 26825169 PMC8491548

[90] Röhrig F Schulze A. The multifaceted roles of fatty acid synthesis in cancer. Nat Rev Cancer. (2016) 16:732–49. 10.1038/nrc.2016.89 27658529

[91] Zhu Z Zhao X Zhao L Yang H Liu L Li J. p54 nrb /NONO regulates lipid metabolism and breast cancer growth through SREBP-1A. Oncogene. (2016) 35:1399–410. 10.1038/onc.2015.197 26148231

[92] Carito V Bonuccelli G Martinez-Outschoorn UE Whitaker-Menezes D Caroleo MC Cione E. Metabolic remodeling of the tumor microenvironment: migration stimulating factor (MSF) reprograms myofibroblasts toward lactate production, fueling anabolic tumor growth. Cell Cycle. (2012) 11:3403–14. 10.4161/cc.21701 22918248 PMC3466551

[93] Guaita-Esteruelas S Gumà J Masana L Borràs J. The peritumoural adipose tissue microenvironment and cancer. The roles of fatty acid binding protein 4 and fatty acid binding protein 5. Mol Cell Endocrinol. (2017) 462:107–18. 10.1016/j.mce.2017.02.002 28163102

[94] Gupta S Roy A Dwarakanath BS. Metabolic cooperation and competition in the tumor microenvironment: implications for therapy. Front Oncol. (2017) 7:68. 10.3389/fonc.2017.00068 28447025 PMC5388702

[95] Ventura R Mordec K Waszczuk J Wang Z Lai J Fridlib M. Inhibition of *de novo* palmitate synthesis by fatty acid synthase induces apoptosis in tumor cells by remodeling cell membranes, inhibiting signaling pathways, and reprogramming gene expression. EBioMedicine. (2015) 2:808–24. 10.1016/j.ebiom.2015.06.020 26425687 PMC4563160

[96] Jiang L Xiao L Sugiura H Huang X Ali A Kuro-o M. Metabolic reprogramming during TGFβ1-induced epithelial-to-mesenchymal transition. Oncogene. (2015) 34:3908–16. 10.1038/onc.2014.321 25284588 PMC4387121

[97] English D Brindley DN Spiegel S Garcia JGN. Lipid mediators of angiogenesis and the signalling pathways they initiate. Biochim Biophys Acta. (2002) 1582:228–39. 10.1016/s1388-1981(02)00176-2 12069833

[98] Wong BW Wang X Zecchin A Thienpont B Cornelissen I Kalucka J. The role of fatty acid β-oxidation in lymphangiogenesis. Nature. (2017) 542:49–54. 10.1038/nature21028 28024299

[99] Uhlén M Fagerberg L Hallström BM Lindskog C Oksvold P Mardinoglu A. Proteomics. Tissue-based map of the human proteome. Science. (2015) 347:1260419. 10.1126/science.1260419 25613900

[100] Uhlen M Zhang C Lee S Sjöstedt E Fagerberg L Bidkhori G. A pathology atlas of the human cancer transcriptome. Science. (2017) 357:eaan2507. 10.1126/science.aan2507 28818916

[101] Uhlén M Björling E Agaton C Szigyarto CA-K Amini B Andersen E. A human protein atlas for normal and cancer tissues based on antibody proteomics. Mol Cell Proteomics. (2005) 4:1920–32. 10.1074/mcp.M500279-MCP200 16127175

[102] Osugi J Yamaura T Muto S Okabe N Matsumura Y Hoshino M. Prognostic impact of the combination of glucose transporter 1 and ATP citrate lyase in node-negative patients with non-small lung cancer. Lung Cancer. (2015) 88:310–8. 10.1016/j.lungcan.2015.03.004 25837797

[103] Csanadi A Kayser C Donauer M Gumpp V Aumann K Rawluk J. Prognostic value of malic enzyme and ATP-citrate lyase in non-small cell lung cancer of the young and the elderly. PLoS ONE. (2015) 10:e0126357. 10.1371/journal.pone.0126357 25962060 PMC4427316

[104] Wen J Min X Shen M Hua Q Han Y Zhao L. ACLY facilitates colon cancer cell metastasis by CTNNB1. J Exp Clin Cancer Res. (2019) 38:401. 10.1186/s13046-019-1391-9 31511060 PMC6740040

[105] Conde E Suarez-Gauthier A García-García E Lopez-Rios F Lopez-Encuentra A García-Lujan R. Specific pattern of LKB1 and phospho-acetyl-CoA carboxylase protein immunostaining in human normal tissues and lung carcinomas. Hum Pathol. (2007) 38:1351–60. 10.1016/j.humpath.2007.01.022 17521700

[106] Bai J Zhang X Kang X Jin L Wang P Wang Z. Screening of core genes and pathways in breast cancer development via comprehensive analysis of multi gene expression datasets. Oncol Lett. (2019) 18:5821–30. 10.3892/ol.2019.10979 31788055 PMC6865771

[107] Luo D Xiao H Dong J Li Y Feng G Cui M. B7-H3 regulates lipid metabolism of lung cancer through SREBP1-mediated expression of FASN. Biochem Biophys Res Commun. (2017) 482:1246–51. 10.1016/j.bbrc.2016.12.021 27939887

[108] Wang H Xi Q Wu G. Fatty acid synthase regulates invasion and metastasis of colorectal cancer via Wnt signaling pathway. Cancer Med. (2016) 5:1599–606. 10.1002/cam4.711 27139420 PMC4864275

[109] Zaytseva YY Rychahou PG Gulhati P Elliott VA Mustain WC O'Connor K. Inhibition of fatty acid synthase attenuates CD44-associated signaling and reduces metastasis in colorectal cancer. Cancer Res. (2012) 72:1504–17. 10.1158/0008-5472.CAN-11-4057 22266115 PMC3596828

[110] Mohamed AH Said NM. Immunohistochemical expression of fatty acid synthase and vascular endothelial growth factor in primary colorectal cancer: a clinicopathological study. J Gastrointest Cancer. (2019) 50:485–92. 10.1007/s12029-018-0104-5 29681001

[111] Ogino S Nosho K Meyerhardt JA Kirkner GJ Chan AT Kawasaki T. Cohort study of fatty acid synthase expression and patient survival in colon cancer. J Clin Oncol. (2008) 26:5713–20. 10.1200/JCO.2008.18.2675 18955444 PMC2630484

[112] Gharib E Nasrinasrabadi P Zali MR. Development and validation of a lipogenic genes panel for diagnosis and recurrence of colorectal cancer. PLoS ONE. (2020) 15:e0229864. 10.1371/journal.pone.0229864 32155177 PMC7064220

[113] Puig T Porta R Colomer R. Fatty acid synthase: a new anti-tumor target. Med Clin. (2009) 132:359–63. 10.1016/j.medcli.2008.07.022 19268984

[114] Giró-Perafita A Sarrats A Pérez-Bueno F Oliveras G Buxó M Brunet J. Fatty acid synthase expression and its association with clinico-histopathological features in triple-negative breast cancer. Oncotarget. (2017) 8:74391–405. 10.18632/oncotarget.20152 29088795 PMC5650350

[115] Corominas-Faja B Vellon L Cuyàs E Buxó M Martin-Castillo B Serra D. Clinical and therapeutic relevance of the metabolic oncogene fatty acid synthase in HER2+ breast cancer. Histol Histopathol. (2017) 32:687–98. 10.14670/HH-11-830 27714708 PMC5784426

[116] Kristiansen G. Immunohistochemical algorithms in prostate diagnostics: what's new? Pathologe. (2009) 30:146–53. 10.1007/s00292-009-1230-4 19795124

[117] Shah US Dhir R Gollin SM Chandran UR Lewis D Acquafondata M. Fatty acid synthase gene overexpression and copy number gain in prostate adenocarcinoma. Hum Pathol. (2006) 37:401–9. 10.1016/j.humpath.2005.11.022 16564913

[118] Myers JS von Lersner AK Sang Q-XA. Proteomic upregulation of fatty acid synthase and fatty acid binding protein 5 and identification of cancer- and race-specific pathway Associations in Human Prostate Cancer Tissues. J Cancer. (2016) 7:1452–64. 10.7150/jca.15860 27471561 PMC4964129

[119] Swinnen JV Vanderhoydonc F Elgamal AA Eelen M Vercaeren I Joniau S. Selective activation of the fatty acid synthesis pathway in human prostate cancer. Int J Cancer. (2000) 88:176–9. 10.1002/1097-0215(20001015)88:2<176::aid-ijc5>3.0.co;2-3 11004665

[120] Vargas T Moreno-Rubio J Herranz J Cejas P Molina S Mendiola M. 3'UTR polymorphism in ACSL1 gene correlates with expression levels and poor clinical outcome in colon cancer patients. PLoS ONE. (2016) 11:e0168423. 10.1371/journal.pone.0168423 27992526 PMC5167383

[121] Chen W-C Wang C-Y Hung Y-H Weng T-Y Yen M-C Lai M-D. Systematic analysis of gene expression alterations and clinical outcomes for long-chain acyl-coenzyme a synthetase family in cancer. PLoS ONE. (2016) 11:e0155660. 10.1371/journal.pone.0155660 27171439 PMC4865206

[122] Wu X Li Y Wang J Wen X Marcus MT Daniels G. Long chain fatty Acyl-CoA synthetase 4 is a biomarker for and mediator of hormone resistance in human breast cancer. PLoS ONE. (2013) 8:e77060. 10.1371/journal.pone.0077060 24155918 PMC3796543

[123] Yen M-C Kan J-Y Hsieh C-J Kuo P-L Hou M-F Hsu Y-L. Association of long-chain acyl-coenzyme a synthetase 5 expression in human breast cancer by estrogen receptor status and its clinical significance. Oncol Rep. (2017) 37:3253–60. 10.3892/or.2017.5610 28498416

[124] Huang J Fan X-X He J Pan H Li R-Z Huang L. SCD1 is associated with tumor promotion, late stage and poor survival in lung adenocarcinoma. Oncotarget. (2016) 7:39970–9. 10.18632/oncotarget.9461 27223066 PMC5129985

[125] Choi S Yoo YJ Kim H Lee H Chung H Nam M-H. Clinical and biochemical relevance of monounsaturated fatty acid metabolism targeting strategy for cancer stem cell elimination in colon cancer. Biochem Biophys Res Commun. (2019) 519:100–5. 10.1016/j.bbrc.2019.08.137 31481234

[126] Holder AM Gonzalez-Angulo AM Chen H Akcakanat A Do K-A Fraser Symmans W. High stearoyl-CoA desaturase 1 expression is associated with shorter survival in breast cancer patients. Breast Cancer Res Treat. (2013) 137:319–27. 10.1007/s10549-012-2354-4 23208590 PMC3556743

[127] Wang D Lin Y Gao B Yan S Wu H Li Y. Reduced expression of fads1 predicts worse prognosis in non-small-cell lung cancer. J Cancer. (2016) 7:1226–32. 10.7150/jca.15403 27390597 PMC4934030

[128] Watkins DN Lenzo JC Segal A Garlepp MJ Thompson PJ. Expression and localization of cyclo-oxygenase isoforms in non-small cell lung cancer. Eur Respir J. (1999) 14:412–8. 10.1034/j.1399-3003.1999.14b28.x 10515422

[129] Yuan A Yu C-J Shun C-T Luh K-T Kuo S-H Lee Y-C. Total cyclooxygenase-2 mRNA levels correlate with vascular endothelial growth factor mRNA levels, tumor angiogenesis and prognosis in non-small cell lung cancer patients. Int J Cancer. (2005) 115:545–55. 10.1002/ijc.20898 15704107

[130] Brabender J Park J Metzger R Schneider PM Lord RV Hölscher AH. Prognostic significance of cyclooxygenase 2 mRNA expression in non-small cell lung cancer. Ann Surg. (2002) 235:440–3. 10.1097/00000658-200203000-00017 11882767 PMC1422451

[131] Soumaoro LT Uetake H Higuchi T Takagi Y Enomoto M Sugihara K. Cyclooxygenase-2 expression: a significant prognostic indicator for patients with colorectal cancer. Clin Cancer Res. (2004) 10:8465–71. 10.1158/1078-0432.CCR-04-0653 15623626

[132] Denkert C Winzer K-J Hauptmann S. Prognostic impact of cyclooxygenase-2 in breast cancer. Clin Breast Cancer. (2004) 4:428–33. 10.3816/cbc.2004.n.006 15023244

[133] Bin W He W Feng Z Xiangdong L Yong C Lele K. Prognostic relevance of cyclooxygenase-2 (COX-2) expression in Chinese patients with prostate cancer. Acta Histochem. (2011) 113:131–6. 10.1016/j.acthis.2009.09.004 19836060

[134] Fan X Weng Y Bai Y Wang Z Wang S Zhu J. Lipin-1 determines lung cancer cell survival and chemotherapy sensitivity by regulation of endoplasmic reticulum homeostasis and autophagy. Cancer Med. (2018) 7:2541–54. 10.1002/cam4.1483 29659171 PMC6010863

[135] Dinarvand N Khanahmad H Hakimian SM Sheikhi A Rashidi B Bakhtiari H. Expression and clinicopathological significance of lipin-1 in human breast cancer and its association with p53 tumor suppressor gene. J Cell Physiol. (2020) 235:5835–46. 10.1002/jcp.29523 31970786

[136] He J Zhang F Tay LWR Boroda S Nian W Levental KR. Lipin-1 regulation of phospholipid synthesis maintains endoplasmic reticulum homeostasis and is critical for triple-negative breast cancer cell survival. FASEB J. (2017) 31:2893–904. 10.1096/fj.201601353R 28347999 PMC6137500

[137] Fujimoto M Yoshizawa A Sumiyoshi S Sonobe M Menju T Hirata M. Adipophilin expression in lung adenocarcinoma is associated with apocrine-like features and poor clinical prognosis: an immunohistochemical study of 328 cases. Histopathology. (2017) 70:232–41. 10.1111/his.13048 27467545

[138] Zhou C Wang M Zhou L Zhang Y Liu W Qin W. Prognostic significance of PLIN1 expression in human breast cancer. Oncotarget. (2016) 7:54488–502. 10.18632/oncotarget.10239 27359054 PMC5342357

[139] Lucenay KS Doostan I Karakas C Bui T Ding Z Mills GB. Cyclin E associates with the lipogenic enzyme ATP-citrate lyase to enable malignant growth of breast cancer cells. Cancer Res. (2016) 76:2406–18. 10.1158/0008-5472.CAN-15-1646 26928812 PMC4873469

[140] Gao C Zhuang J Li H Liu C Zhou C Liu L. Development of a risk scoring system for evaluating the prognosis of patients with Her2-positive breast cancer. Cancer Cell Int. (2020) 20:121. 10.1186/s12935-020-01175-1 32322168 PMC7161270

[141] Enciu A-M Radu E Popescu ID Hinescu ME Ceafalan LC. Targeting CD36 as biomarker for metastasis prognostic: how far from translation into clinical practice? Biomed Res Int. (2018) 2018:7801202. 10.1155/2018/7801202 30069479 PMC6057354

[142] Cha YJ Kim HM Koo JS. Expression of lipid metabolism-related proteins differs between invasive lobular carcinoma and invasive ductal carcinoma. Int J Mol Sci. (2017) 18:232. 10.3390/ijms18010232 28124996 PMC5297861

[143] Aiderus A Black MA Dunbier AK. Fatty acid oxidation is associated with proliferation and prognosis in breast and other cancers. BMC Cancer. (2018) 18:805. 10.1186/s12885-018-4626-9 30092766 PMC6085695

[144] Saraon P Trudel D Kron K Dmitromanolakis A Trachtenberg J Bapat B. Evaluation and prognostic significance of ACAT1 as a marker of prostate cancer progression. Prostate. (2014) 74:372–80. 10.1002/pros.22758 24311408

[145] Saraon P Cretu D Musrap N Karagiannis GS Batruch I Drabovich AP. Quantitative proteomics reveals that enzymes of the ketogenic pathway are associated with prostate cancer progression. Mol Cell Proteomics. (2013) 12:1589–601. 10.1074/mcp.M112.023887 23443136 PMC3675816

[146] Chen S-W Chou C-T Chang C-C Li Y-J Chen S-T Lin I-C. HMGCS2 enhances invasion and metastasis via direct interaction with PPARα to activate Src signaling in colorectal cancer and oral cancer. Oncotarget. (2017) 8:22460–76. 10.18632/oncotarget.13006 27816970 PMC5410236

[147] Wan S Xi M Zhao H-B Hua W Liu Y-L Zhou Y-L. HMGCS2 functions as a tumor suppressor and has a prognostic impact in prostate cancer. Pathol Res Pract. (2019) 215:152464. 10.1016/j.prp.2019.152464 31176575

[148] Bengtsson E Nerjovaj P Wangefjord S Nodin B Eberhard J Uhlén M. HMG-CoA reductase expression in primary colorectal cancer correlates with favourable clinicopathological characteristics and an improved clinical outcome. Diagn Pathol. (2014) 9:78. 10.1186/1746-1596-9-78 24708688 PMC4000148

[149] Kim H Seol YM Choi YJ Shin H-J Chung JS Shin N. HMG CoA reductase expression as a prognostic factor in korean patients with breast cancer: a retrospective study. Medicine. (2019) 98:e14968. 10.1097/MD.0000000000014968 30921201 PMC6456116

[150] Borgquist S Jögi A Pontén F Rydén L Brennan DJ Jirström K. Prognostic impact of tumour-specific HMG-CoA reductase expression in primary breast cancer. Breast Cancer Res. (2008) 10:R79. 10.1186/bcr2146 18808688 PMC2614512

[151] Aguirre-Portolés C Feliu J Reglero G Ramírez de Molina A. ABCA1 overexpression worsens colorectal cancer prognosis by facilitating tumour growth and caveolin-1-dependent invasiveness, and these effects can be ameliorated using the BET inhibitor apabetalone. Mol Oncol. (2018) 12:1735–52. 10.1002/1878-0261.12367 30098223 PMC6166002

[152] Yang G Wang X-J Huang L-J Zhou Y-A Tian F Zhao J-B. High ABCG4 expression is associated with poor prognosis in non-small-cell lung cancer patients treated with cisplatin-based chemotherapy. PLoS ONE. (2015) 10:e0135576. 10.1371/journal.pone.0135576 26270652 PMC4535915

[153] Hostettler L Zlobec I Terracciano L Lugli A. ABCG5-positivity in tumor buds is an indicator of poor prognosis in node-negative colorectal cancer patients. World J Gastroenterol. (2010) 16:732–9. 10.3748/wjg.v16.i6.732 20135722 PMC2817062

[154] Caruso MG Osella AR Notarnicola M Berloco P Leo S Bonfiglio C. Prognostic value of low density lipoprotein receptor expression in colorectal carcinoma. Oncol Rep. (1998) 5:927–30. 10.3892/or.5.4.927 9625848

[155] Zhang N Zhang H Liu Y Su P Zhang J Wang X. SREBP1, targeted by miR-18a-5p, modulates epithelial-mesenchymal transition in breast cancer via forming a co-repressor complex with snail and HDAC1/2. Cell Death Differ. (2019) 26:843–59. 10.1038/s41418-018-0158-8 29988076 PMC6461794

[156] Sharma B Gupta V Dahiya D Kumar H Vaiphei K Agnihotri N. Clinical relevance of cholesterol homeostasis genes in colorectal cancer. Biochim Biophys Acta Mol Cell Biol Lipids. (2019) 1864:1314–27. 10.1016/j.bbalip.2019.06.008 31202724

[157] Li X Wu JB Li Q Shigemura K Chung LWK Huang W-C SREBP-2 promotes stem cell-like properties and metastasis by transcriptional activation of c-Myc in prostate cancer. Oncotarget. (2016) 7:12869–84. 10.18632/oncotarget.7331 26883200 PMC4914327

[158] Hazra S Peebles KA Sharma S Mao JT Dubinett SM. The role of ppargamma in the cyclooxygenase pathway in lung cancer. PPAR Res. (2008) 2008:790568. 10.1155/2008/790568 18769553 PMC2526169

[159] Sasaki H Tanahashi M Yukiue H Moiriyama S Kobayashi Y Nakashima Y. Decreased perioxisome proliferator-activated receptor gamma gene expression was correlated with poor prognosis in patients with lung cancer. Lung Cancer. (2002) 36:71–6. 10.1016/s0169-5002(01)00449-4 11891036

[160] Ogino S Shima K Baba Y Nosho K Irahara N Kure S. Colorectal cancer expression of peroxisome proliferator-activated receptor gamma (PPARG, PPARgamma) is associated with good prognosis. Gastroenterology. (2009) 136:1242–50. 10.1053/j.gastro.2008.12.048 19186181 PMC2663601

[161] Abduljabbar R Al-Kaabi MM Negm OH Jerjees D Muftah AA Mukherjee A. Prognostic and biological significance of peroxisome proliferator-activated receptor-gamma in luminal breast cancer. Breast Cancer Res Treat. (2015) 150:511–22. 10.1007/s10549-015-3348-9 25794775

[162] Jiang Y Zou L Zhang C He S Cheng C Xu J. PPARgamma and Wnt/beta-Catenin pathway in human breast cancer: expression pattern, molecular interaction and clinical/prognostic correlations. J Cancer Res Clin Oncol. (2009) 135:1551–9. 10.1007/s00432-009-0602-8 19495794 PMC12160272

[163] Nakamura Y Suzuki T Sugawara A Arai Y Sasano H. Peroxisome proliferator-activated receptor gamma in human prostate carcinoma. Pathol Int. (2009) 59:288–93. 10.1111/j.1440-1827.2009.02367.x 19432669

[164] Melloni G Muriana P Bandiera A Fontana R Maggioni D Russo V. Prognostic role of liver X receptor-alpha in resected stage II and III non-small-cell lung cancer. Clin Respir J. (2018) 12:241–6. 10.1111/crj.12522 27401614

[165] Jin X Zhang K-J Guo X Myers R Ye Z Zhang Z-P. Fatty acid synthesis pathway genetic variants and clinical outcome of non-small cell lung cancer patients after surgery. Asian Pac J Cancer Prev. (2014) 15:7097–103. 10.7314/apjcp.2014.15.17.7097 25227797

[166] Xie S Zhou F Wang J Cao H Chen Y Liu X. Functional polymorphisms of ATP citrate lyase gene predicts clinical outcome of patients with advanced colorectal cancer. World J Surg Oncol. (2015) 13:42. 10.1186/s12957-015-0440-x 25890184 PMC4359538

[167] Zulato E Bergamo F De Paoli A Griguolo G Esposito G De Salvo GL. Prognostic significance of AMPK activation in advanced stage colorectal cancer treated with chemotherapy plus bevacizumab. Br J Cancer. (2014) 111:25–32. 10.1038/bjc.2014.274 24892446 PMC4090737

[168] O'Malley J Kumar R Kuzmin AN Pliss A Yadav N Balachandar S. Lipid quantification by Raman microspectroscopy as a potential biomarker in prostate cancer. Cancer Lett. (2017) 397:52–60. 10.1016/j.canlet.2017.03.025 28342983 PMC5449194

[169] Merino Salvador M Gómez de Cedrón M Moreno Rubio J Falagán Martínez S Sánchez Martínez R Casado E. Lipid metabolism and lung cancer. Crit Rev Oncol Hematol. (2017) 112:31–40. 10.1016/j.critrevonc.2017.02.001 28325263

[170] Cerne D Zitnik IP Sok M. Increased fatty acid synthase activity in non-small cell lung cancer tissue is a weaker predictor of shorter patient survival than increased lipoprotein lipase activity. Arch Med Res. (2010) 41:405–9. 10.1016/j.arcmed.2010.08.007 21044743

[171] Notarnicola M Tutino V Calvani M Lorusso D Guerra V Caruso MG. Serum levels of fatty acid synthase in colorectal cancer patients are associated with tumor stage. J Gastrointest Cancer. (2012) 43:508–511. 10.1007/s12029-011-9300-2 21727995

[172] Long Q Yi Y Qiu J Xu C Huang P. Fatty acid synthase (FASN) levels in serum of colorectal cancer patients: correlation with clinical outcomes. Tumour Biol. (2014) 35:3855–9. 10.1007/s13277-013-1510-8 24430360

[173] Bian Y Yu Y Wang S Li L. Up-regulation of fatty acid synthase induced by EGFR/ERK activation promotes tumor growth in pancreatic cancer. Biochem Biophys Res Commun. (2015) 463:612–7. 10.1016/j.bbrc.2015.05.108 26043686

[174] Padanad MS Konstantinidou G Venkateswaran N Melegari M Rindhe S Mitsche M. Fatty acid oxidation mediated by Acyl-CoA synthetase long chain 3 is required for mutant kras lung tumorigenesis. Cell Rep. (2016) 16:1614–28. 10.1016/j.celrep.2016.07.009 27477280 PMC4981512

[175] Wang J Scholtens D Holko M Ivancic D Lee O Hu H. Lipid metabolism genes in contralateral unaffected breast and estrogen receptor status of breast cancer. Cancer Prev Res. (2013) 6:321–30. 10.1158/1940-6207.CAPR-12-0304 23512947

[176] Obinata D Takayama K Fujiwara K Suzuki T Tsutsumi S Fukuda N. Targeting Oct1 genomic function inhibits androgen receptor signaling and castration-resistant prostate cancer growth. Oncogene. (2016) 35:6350–8. 10.1038/onc.2016.171 27270436

[177] Xia H Lee KW Chen J Kong SN Sekar K Deivasigamani A. Simultaneous silencing of ACSL4 and induction of GADD45B in hepatocellular carcinoma cells amplifies the synergistic therapeutic effect of aspirin and sorafenib. Cell Death Discov. (2017) 3:17058. 10.1038/cddiscovery.2017.58 28900541 PMC5592242

[178] Tang Y Zhou J Hooi SC Jiang Y-M Lu G-D. Fatty acid activation in carcinogenesis and cancer development: essential roles of long-chain acyl-CoA synthetases. Oncol Lett. (2018) 16:1390–6. 10.3892/ol.2018.8843 30008815 PMC6036470

[179] Takeuchi K Reue K. Biochemistry, physiology, and genetics of GPAT, AGPAT, and lipin enzymes in triglyceride synthesis. Am J Physiol Endocrinol Metab. (2009) 296:E1195–209. 10.1152/ajpendo.90958.2008 19336658 PMC2692402

[180] Marchan R Büttner B Lambert J Edlund K Glaeser I Blaszkewicz M. Glycerol-3-phosphate acyltransferase 1 promotes tumor cell migration and poor survival in ovarian carcinoma. Cancer Res. (2017) 77:4589–601. 10.1158/0008-5472.CAN-16-2065 28652252

[181] Niesporek S Denkert C Weichert W Köbel M Noske A Sehouli J. Expression of lysophosphatidic acid acyltransferase beta (LPAAT-beta) in ovarian carcinoma: correlation with tumour grading and prognosis. Br J Cancer. (2005) 92:1729–36. 10.1038/sj.bjc.6602528 15841084 PMC2362024

[182] Roe ND Handzlik MK Li T Tian R. The role of diacylglycerol acyltransferase (DGAT) 1 and 2 in cardiac metabolism and function. Sci Rep. (2018) 8:4983. 10.1038/s41598-018-23223-7 29563512 PMC5862879

[183] Xin C Chu L Zhang L Geng D Wang Y Sun D. Expression of cytosolic phospholipase A2 (cPLA2)-Arachidonic acid (AA)-Cyclooxygenase-2 (COX-2) pathway factors in lung cancer patients and its implication in lung cancer early detection and prognosis. Med Sci Monit. (2019) 25:5543–51. 10.12659/MSM.915314 31347609 PMC6679621

[184] Church RD Yu J Fleshman JW Shannon WD Govindan R McLeod HL. RNA profiling of cyclooxygenases 1 and 2 in colorectal cancer. Br J Cancer. (2004) 91:1015–8. 10.1038/sj.bjc.6602119 15328521 PMC2747709

[185] Tury S Becette V Assayag F Vacher S Benoist C Kamal M. Combination of COX-2 expression and PIK3CA mutation as prognostic and predictive markers for celecoxib treatment in breast cancer. Oncotarget. (2016) 7:85124–41. 10.18632/oncotarget.13200 27835884 PMC5356723

[186] Frank B Hoffmeister M Klopp N Illig T Chang-Claude J Brenner H. Polymorphisms in inflammatory pathway genes and their association with colorectal cancer risk. Int J Cancer. (2010) 127:2822–30. 10.1002/ijc.25299 21351261

[187] Coghill AE Newcomb PA Poole EM Hutter CM Makar KW Duggan D. Genetic variation in inflammatory pathways is related to colorectal cancer survival. Clin Cancer Res. (2011) 17:7139–47. 10.1158/1078-0432.CCR-11-1134 21976545 PMC3218294

[188] Hao Y Li D Xu Y Ouyang J Wang Y Zhang Y. Investigation of lipid metabolism dysregulation and the effects on immune microenvironments in pan-cancer using multiple omics data. BMC Bioinform. (2019) 20:195. 10.1186/s12859-019-2734-4 31074374 PMC6509864

[189] de Gonzalo-Calvo D López-Vilaró L Nasarre L Perez-Olabarria M Vázquez T Escuin D. Intratumor cholesteryl ester accumulation is associated with human breast cancer proliferation and aggressive potential: a molecular and clinicopathological study. BMC Cancer. (2015) 15:460. 10.1186/s12885-015-1469-5 26055977 PMC4460760

[190] Brennan DJ Laursen H O'Connor DP Borgquist S Uhlen M Gallagher WM. Tumor-specific HMG-CoA reductase expression in primary premenopausal breast cancer predicts response to tamoxifen. Breast Cancer Res. (2011) 13:R12. 10.1186/bcr2820 21281480 PMC3109580

[191] Butt S Butt T Jirström K Hartman L Amini R-M Zhou W. The target for statins, HMG-CoA reductase, is expressed in ductal carcinoma-in situ and may predict patient response to radiotherapy. Ann Surg Oncol. (2014) 21:2911–9. 10.1245/s10434-014-3708-4 24777857

[192] Bjarnadottir O Romero Q Bendahl P-O Jirström K Rydén L Loman N. Targeting HMG-CoA reductase with statins in a window-of-opportunity breast cancer trial. Breast Cancer Res Treat. (2013) 138:499–508. 10.1007/s10549-013-2473-6 23471651

[193] Schimanski S Wild PJ Treeck O Horn F Sigruener A Rudolph C. Expression of the lipid transporters ABCA3 and ABCA1 is diminished in human breast cancer tissue. Horm Metab Res. (2010) 42:102–9. 10.1055/s-0029-1241859 19902402

[194] Wang Y Liu H Ready NE Su L Wei Y Christiani DC. Genetic variants in ABCG1 are associated with survival of nonsmall-cell lung cancer patients. Int J Cancer. (2016) 138:2592–601. 10.1002/ijc.29991 26757251 PMC5294935

[195] Zhou T Zhan J Fang W Zhao Y Yang Y Hou X. Serum low-density lipoprotein and low-density lipoprotein expression level at diagnosis are favorable prognostic factors in patients with small-cell lung cancer (SCLC). BMC Cancer. (2017) 17:269. 10.1186/s12885-017-3239-z 28410578 PMC5391547

[196] Sun Y He W Luo M Zhou Y Chang G Ren W. SREBP1 regulates tumorigenesis and prognosis of pancreatic cancer through targeting lipid metabolism. Tumour Biol. (2015) 36:4133–41. 10.1007/s13277-015-3047-5 25589463

[197] Grygiel-Górniak B. Peroxisome proliferator-activated receptors and their ligands: nutritional and clinical implications – a review. Nutr J. (2014) 13:17. 10.1186/1475-2891-13-17 24524207 PMC3943808

[198] Shao W Kuhn C Mayr D Ditsch N Kailuwait M Wolf V. Cytoplasmic PPARγ is a marker of poor prognosis in patients with Cox-1 negative primary breast cancers. J Transl Med. (2020) 18:94. 10.1186/s12967-020-02271-6 32085795 PMC7035771

[199] Liang X Fan X Tan K Zhang L Jian L Yu L. Peroxisome proliferators-activated receptor gamma polymorphisms and colorectal cancer risk. J Cancer Res Ther. (2018) 14:S306–10. 10.4103/0973-1482.235346 29970681

[200] Sebio A Gerger A Matsusaka S Yang D Zhang W Stremitzer S. Genetic variants within obesity-related genes are associated with tumor recurrence in patients with stages II/III colon cancer. Pharmacogenet Genomics. (2015) 25:30–7. 10.1097/FPC.0000000000000101 25379721 PMC4260998

[201] Bauerschlag DO Maass N Leonhardt P Verburg FA Pecks U Zeppernick F. Fatty acid synthase overexpression: target for therapy and reversal of chemoresistance in ovarian cancer. J Transl Med. (2015) 13:146. 10.1186/s12967-015-0511-3 25947066 PMC4504229

[202] Malvi P Chaube B Pandey V Vijayakumar MV Boreddy PR Mohammad N. Obesity induced rapid melanoma progression is reversed by orlistat treatment and dietary intervention: role of adipokines. Mol Oncol. (2015) 9:689–703. 10.1016/j.molonc.2014.11.006 25499031 PMC5528703

[203] Konkel B Caflisch L Diaz Duque AE Brenner A Updated results from a prospective, randomized phase 2 study in patients with first relapse of high-grade astrocytoma using TVB-2640 in combination with avastin versus avastin alone. NeuroOncology. (2018) 20:vi16 10.1093/NEUONC/NOY148.058

[204] Dean EJ Falchook GS Patel MR Brenner AJ Infante JR Arkenau HT Preliminary activity in the first in human study of the first-in-class fatty acid synthase (FASN) inhibitor, TVB-2640. J Clin Oncol. (2017) 34:2512 10.1200/JCO.2016.34.15_suppl.2512 PMC8040281 33870151

[205] Luo J Hong Y Lu Y Qiu S Chaganty BK Zhang L. Acetyl-CoA carboxylase rewires cancer metabolism to allow cancer cells to survive inhibition of the warburg effect by cetuximab. Cancer Lett. (2017) 384:39–49. 10.1016/j.canlet.2016.09.020 27693630 PMC5110372

[206] Svensson RU Parker SJ Eichner LJ Kolar MJ Wallace M Brun SN. Inhibition of acetyl-CoA carboxylase suppresses fatty acid synthesis and tumor growth of non-small-cell lung cancer in preclinical models. Nat Med. (2016) 22:1108–19. 10.1038/nm.4181 27643638 PMC5053891

[207] Koltun DO Zilbershtein TM Migulin VA Vasilevich NI Parkhill EQ Glushkov AI. Potent, orally bioavailable, liver-selective stearoyl-CoA desaturase (SCD) inhibitors. Bioorg Med Chem Lett. (2009) 19:4070–4. 10.1016/j.bmcl.2009.06.017 19577469

[208] Ma MKF Lau EYT Leung DHW Lo J Ho NPY Cheng LKW. Stearoyl-CoA desaturase regulates sorafenib resistance via modulation of ER stress-induced differentiation. J Hepatol. (2017) 67:979–90. 10.1016/j.jhep.2017.06.015 28647567

[209] Potze L di Franco S Kessler JH Stassi G Medema JP. Betulinic acid kills colon cancer stem cells. Curr Stem Cell Res Ther. (2016) 11:427–33. 10.1038/onc.2015.102 26647913

[210] Wang H Dong F Wang Y Wang X Hong D Liu Y. Betulinic acid induces apoptosis of gallbladder cancer cells via repressing SCD1. Acta Biochim Biophys Sin. (2020) 52:200–6. 10.1093/abbs/gmz148 31915810

[211] Pisanu ME Noto A De Vitis C Morrone S Scognamiglio G Botti G. Blockade of stearoyl-CoA-desaturase 1 activity reverts resistance to cisplatin in lung cancer stem cells. Cancer Lett. (2017) 406:93–104. 10.1016/j.canlet.2017.07.027 28797843

[212] Noto A Raffa S De Vitis C Roscilli G Malpicci D Coluccia P. Stearoyl-CoA desaturase-1 is a key factor for lung cancer-initiating cells. Cell Death Dis. (2013) 4:e947. 10.1038/cddis.2013.444 24309934 PMC3877537

[213] von Roemeling CA Marlow LA Wei JJ Cooper SJ Caulfield TR Wu K. Stearoyl-CoA desaturase 1 is a novel molecular therapeutic target for clear cell renal cell carcinoma. Clin Cancer Res. (2013) 19:2368–80. 10.1158/1078-0432.CCR-12-3249 23633458 PMC3644999

[214] Samudio I Harmancey R Fiegl M Kantarjian H Konopleva M Korchin B. Pharmacologic inhibition of fatty acid oxidation sensitizes human leukemia cells to apoptosis induction. J Clin Invest. (2010) 120:142–56. 10.1172/JCI38942 20038799 PMC2799198

[215] Schlaepfer IR Rider L Rodrigues LU Gijon MA Pac CT Romero L. Lipid catabolism via CPT1 as a therapeutic target for prostate cancer. Mol Cancer Ther. (2014) 13:2361–71. 10.1158/1535-7163.MCT-14-0183 25122071 PMC4185227

[216] Lin H Patel S Affleck VS Wilson I Turnbull DM Joshi AR. Fatty acid oxidation is required for the respiration and proliferation of malignant glioma cells. Neuro Oncol. (2017) 19:43–54. 10.1093/neuonc/now128 27365097 PMC5193020

[217] Flaig TW Salzmann-Sullivan M Su LJ Zhang Z Joshi M Gijon MA. Lipid catabolism inhibition sensitizes prostate cancer cells to antiandrogen blockade. Oncotarget. (2017) 8:56051–65. 10.18632/oncotarget.17359 28915573 PMC5593544

[218] Liu PP Liu J Jiang WQ Carew JS Ogasawara MA Pelicano H. Elimination of chronic lymphocytic leukemia cells in stromal microenvironment by targeting CPT with an antiangina drug perhexiline. Oncogene. (2016) 35:5663–73. 10.1038/onc.2016.103 27065330 PMC5064824

[219] Ren XR Wang J Osada T Mook RA Jr Morse MA Barak LS. Perhexiline promotes HER3 ablation through receptor internalization and inhibits tumor growth. Breast Cancer Res. (2015) 17:20. 10.1186/s13058-015-0528-9 25849870 PMC4358700

[220] Camarda R Zhou AY Kohnz RA Balakrishnan S Mahieu C Anderton B. Inhibition of fatty acid oxidation as a therapy for MYC-overexpressing triple-negative breast cancer. Nat Med. (2016) 22:427–32. 10.1038/nm.4055 26950360 PMC4892846

[221] Li N Zhou ZS Shen Y Xu J Miao HH Xiong Y. Inhibition of the sterol regulatory element-binding protein pathway suppresses hepatocellular carcinoma by repressing inflammation in mice. Hepatology. (2017) 65:1936–47. 10.1002/hep.29018 28027595

[222] Soica C Dehelean C Danciu C Wang HM Wenz G Ambrus R. Betulin complex in gamma-cyclodextrin derivatives: properties and antineoplasic activities in *in vitro* and *in vivo* tumor models. Int J Mol Sci. (2012) 13:14992–5011. 10.3390/ijms131114992 23203108 PMC3509624

[223] Li X Wu JB Chung LW Huang WC. Anti-cancer efficacy of SREBP inhibitor, alone or in combination with docetaxel, in prostate cancer harboring p53 mutations. Oncotarget. (2015) 6:41018–32. 10.18632/oncotarget.5879 26512780 PMC4747386

[224] Li X Chen YT Hu P Huang WC. Fatostatin displays high antitumor activity in prostate cancer by blocking SREBP-regulated metabolic pathways and androgen receptor signaling. Mol Cancer Ther. (2014) 13:855–66. 10.1158/1535-7163.MCT-13-0797 24493696 PMC4084917

[225] Williams KJ Argus JP Zhu Y Wilks MQ Marbois BN York AG. An essential requirement for the SCAP/SREBP signaling axis to protect cancer cells from lipotoxicity. Cancer Res. (2013) 73:2850–62. 10.1158/0008-5472.CAN-13-0382-T 23440422 PMC3919498

[226] Kamisuki S Mao Q Abu-Elheiga L Gu Z Kugimiya A Kwon Y. A small molecule that blocks fat synthesis by inhibiting the activation of SREBP. Chem Biol. (2009) 16:882–92. 10.1016/j.chembiol.2009.07.007 19716478

[227] Ceroi A Masson D Roggy A Roumier C Chague C Gauthier T. LXR agonist treatment of blastic plasmacytoid dendritic cell neoplasm restores cholesterol efflux and triggers apoptosis. Blood. (2016) 128:2694–707. 10.1182/blood-2016-06-724807 27702801 PMC5271175

[228] Nguyen TTT Ishida CT Shang E Shu C Torrini C Zhang Y. Activation of LXRbeta inhibits tumor respiration and is synthetically lethal with Bcl-xL inhibition. EMBO Mol Med. (2019) 11:e10769. 10.15252/emmm.201910769 31468706 PMC6783693

[229] Guo D Reinitz F Youssef M Hong C Nathanson D Akhavan D. An LXR agonist promotes glioblastoma cell death through inhibition of an EGFR/AKT/SREBP-1/LDLR-dependent pathway. Cancer Discov. (2011) 1:442–56. 10.1158/2159-8290.CD-11-0102 22059152 PMC3207317

[230] Lee SS Li J Tai JN Ratliff TL Park K Cheng JX. Avasimibe encapsulated in human serum albumin blocks cholesterol esterification for selective cancer treatment. ACS Nano. (2015) 9:2420–32. 10.1021/nn504025a 25662106 PMC5909415

[231] Liu JY Fu WQ Zheng XJ Li W Ren LW Wang JH. Avasimibe exerts anticancer effects on human glioblastoma cells via inducing cell apoptosis and cell cycle arrest. Acta Pharmacol Sin. (2020). 10.1038/s41401-020-0404-8 32451414 PMC7921416

[232] Bandyopadhyay S Li J Traer E Tyner JW Zhou A Oh ST. Cholesterol esterification inhibition and imatinib treatment synergistically inhibit growth of BCR-ABL mutation-independent resistant chronic myelogenous leukemia. PLoS ONE. (2017) 12:e0179558. 10.1371/journal.pone.0179558 28719608 PMC5515395

[233] Watt MJ Clark AK Selth LA Haynes VR Lister N Rebello R. Suppressing fatty acid uptake has therapeutic effects in preclinical models of prostate cancer. Sci Transl Med. (2019) 11:eaau5758. 10.1126/scitranslmed.aau5758 30728288

[234] Longo J Hamilton RJ Masoomian M Khurram N Branchard E Mullen PJ. A pilot window-of-opportunity study of preoperative fluvastatin in localized prostate cancer. Prostate Cancer Prostatic Dis. (2020). 10.1038/s41391-020-0221-7 32203069 PMC7655503

[235] Singh PP Lemanu DP Soop M Bissett IP Harrison J Hill AG. Perioperative simvastatin therapy in major colorectal surgery: a prospective, double-blind randomized controlled trial. J Am Coll Surg. (2016) 223:308–20 e1. 10.1016/j.jamcollsurg.2016.04.004 27086089

[236] Han JY Lee SH Yoo NJ Hyung LS Moon YJ Yun T. A randomized phase II study of gefitinib plus simvastatin versus gefitinib alone in previously treated patients with advanced non-small cell lung cancer. Clin Cancer Res. (2011) 17:1553–60. 10.1158/1078-0432.CCR-10-2525 21411446

[237] Pagano E Borrelli F Orlando P Romano B Monti M Morbidelli L. Pharmacological inhibition of MAGL attenuates experimental colon carcinogenesis. Pharmacol Res. (2017) 119:227–36. 10.1016/j.phrs.2017.02.002 28193521

[238] Liu R Zheng H Li W Guo Q He S Hirasaki Y. Anti-tumor enhancement of Fei-Liu-Ping ointment in combination with celecoxib via cyclooxygenase-2-mediated lung metastatic inflammatory microenvironment in Lewis lung carcinoma xenograft mouse model. J Transl Med. (2015) 13:366. 10.1186/s12967-015-0728-1 26597177 PMC4656184

[239] Suri A Sheng X Schuler KM Zhong Y Han X Jones HM. The effect of celecoxib on tumor growth in ovarian cancer cells and a genetically engineered mouse model of serous ovarian cancer. Oncotarget. (2016) 7:39582–94. 10.18632/oncotarget.8659 27074576 PMC5129955

[240] Marks EI Yee NS. Molecular genetics and targeted therapeutics in biliary tract carcinoma. World J Gastroenterol. (2016) 22:1335–47. 10.3748/wjg.v22.i4.1335 26819503 PMC4721969

[241] Wang Y Zhu M Yuan B Zhang K Zhong M Yi W. VSP-17, a new ppargamma agonist, suppresses the metastasis of triple-negative breast cancer via upregulating the expression of E-Cadherin. Molecules. (2018) 23:121. 10.3390/molecules23010121 29316690 PMC6017286

[242] Laouirem S Sannier A Norkowski E Cauchy F Doblas S Rautou PE. Endothelial fatty liver binding protein 4: a new targetable mediator in hepatocellular carcinoma related to metabolic syndrome. Oncogene. (2019) 38:3033–3046. 10.1038/s41388-018-0597-1 30575815 PMC6484689

[243] Huang M Narita S Inoue T Koizumi A Saito M Tsuruta H. Fatty acid binding protein 4 enhances prostate cancer progression by upregulating matrix metalloproteinases and stromal cell cytokine production. Oncotarget. (2017) 8:111780–94. 10.18632/oncotarget.22908 29340091 PMC5762359

[244] Al-Jameel W Gou X Forootan SS Al Fayi MS Rudland PS Forootan FS. Inhibitor SBFI26 suppresses the malignant progression of castration-resistant PC3-M cells by competitively binding to oncogenic FABP5. Oncotarget. (2017) 8:31041–56. 10.18632/oncotarget.16055 28415688 PMC5458187

[245] Kohlmeier M De Caterina R Ferguson LR Görman U Allayee H Prasad C. Guide and position of the International Society of Nutrigenetics/Nutrigenomics on personalized nutrition: part 2 - ethics, challenges and endeavors of precision nutrition. J Nutrigenet Nutrigenomics. (2016) 9:28–46. 10.1159/000446347 27286972

[246] Dienstmann R Vermeulen L Guinney J Kopetz S Tejpar S Tabernero J Consensus molecular subtypes and the evolution of precision medicine in colorectal cancer. Nat Rev Cancer. (2017) 17:79–92. 10.1038/nrc.2016.126 28050011

[247] Ramos-Lopez O Roman S Martinez-Lopez E Fierro NA Gonzalez-Aldaco K Jose-Abrego A. CD36 genetic variation, fat intake and liver fibrosis in chronic hepatitis C virus infection. World J Hepatol. (2016) 8:1067–74. 10.4254/wjh.v8.i25.1067 27660673 PMC5026998

[248] Carpene C Gomez-Zorita S Deleruyelle S Carpene MA. Novel strategies for preventing diabetes and obesity complications with natural polyphenols. Curr Med Chem. (2015) 22:150–64. 10.2174/0929867321666140815124052 25139462

[249] Farhat G Drummond S Al-Dujaili EAS. Polyphenols and their role in obesity management: a systematic review of randomized clinical trials. Phytother Res. (2017) 31:1005–18. 10.1002/ptr.5830 28493374

[250] Novotny JA Chen T-Y Terekhov AI Gebauer SK Baer DJ Ho L. The effect of obesity and repeated exposure on pharmacokinetic response to grape polyphenols in humans. Mol Nutr Food Res. (2017) 61:11. 10.1002/mnfr.201700043 28654207 PMC5668187

[251] Jiao X Wang Y Lin Y Lang Y Li E Zhang X. Blueberry polyphenols extract as a potential prebiotic with anti-obesity effects on C57BL/6 J mice by modulating the gut microbiota. J Nutr Biochem. (2019) 64:88–100. 10.1016/j.jnutbio.2018.07.008 30471564

[252] Yubero-Serrano EM Gonzalez-Guardia L Rangel-Zuñiga O Delgado-Lista J Gutierrez-Mariscal FM Perez-Martinez P. Mediterranean diet supplemented with coenzyme Q10 modifies the expression of proinflammatory and endoplasmic reticulum stress-related genes in elderly men and women. J Gerontol A Biol Sci Med Sci. (2012) 67:3–10. 10.1093/gerona/glr167 22016358

[253] Lopez-Moreno J Quintana-Navarro GM Delgado-Lista J Garcia-Rios A Alcala-Diaz JF Gomez-Delgado F. mediterranean diet supplemented with coenzyme q10 modulates the postprandial metabolism of advanced glycation end products in elderly men and women. J Gerontol A Biol Sci Med Sci. (2018) 73:340–6. 10.1093/gerona/glw214 28329789

[254] Bayerdörffer E Mannes GA Richter WO Ochsenkühn T Seeholzer G Köpcke W. Decreased high-density lipoprotein cholesterol and increased low-density cholesterol levels in patients with colorectal adenomas. Ann Intern Med. (1993) 118:481–7. 10.7326/0003-4819-118-7-199304010-00001 8442619

[255] Morad SAF Madigan JP Levin JC Abdelmageed N Karimi R Rosenberg DW. Tamoxifen magnifies therapeutic impact of ceramide in human colorectal cancer cells independent of p53. Biochem Pharmacol. (2013) 85:1057–65. 10.1016/j.bcp.2013.01.015 23353700 PMC3604153

[256] Varela LM Ortega-Gomez A Lopez S Abia R Muriana FJG Bermudez B. The effects of dietary fatty acids on the postprandial triglyceride-rich lipoprotein/apoB48 receptor axis in human monocyte/macrophage cells. J Nutr Biochem. (2013) 24:2031–9. 10.1016/j.jnutbio.2013.07.004 24231096

[257] Huerta AE Prieto-Hontoria PL Fernández-Galilea M Escoté X Martínez JA Moreno-Aliaga MJ. Effects of dietary supplementation with EPA and/or α-lipoic acid on adipose tissue transcriptomic profile of healthy overweight/obese women following a hypocaloric diet. Biofactors. (2017) 43:117–31. 10.1002/biof.1317 27507611

[258] Boschmann M Thielecke F. The effects of epigallocatechin-3-gallate on thermogenesis and fat oxidation in obese men: a pilot study. J Am Coll Nutr. (2007) 26:389S–95S. 10.1080/07315724.2007.10719627 17906192

[259] Cardoso GA Salgado JM Cesar Mde C Donado-Pestana CM The effects of green tea consumption and resistance training on body composition and resting metabolic rate in overweight or obese women. J Med Food. (2013) 16:120–7. 10.1089/jmf.2012.0062 23140132

[260] Chen IJ Liu CY Chiu JP Hsu CH. Therapeutic effect of high-dose green tea extract on weight reduction: a randomized, double-blind, placebo-controlled clinical trial. Clin Nutr. (2016) 35:592–9. 10.1016/j.clnu.2015.05.003 26093535

[261] Santamarina AB Carvalho-Silva M Gomes LM Okuda MH Santana AA Streck EL. Decaffeinated green tea extract rich in epigallocatechin-3-gallate prevents fatty liver disease by increased activities of mitochondrial respiratory chain complexes in diet-induced obesity mice. J Nutr Biochem. (2015) 26:1348–56. 10.1016/j.jnutbio.2015.07.002 26300331

[262] Huang J Feng S Liu A Dai Z Wang H Reuhl K Green tea polyphenol EGCG alleviates metabolic abnormality and fatty liver by decreasing bile acid and lipid absorption in mice. Mol Nutr Food Res. (2018) 62 10.1002/mnfr.201700696 PMC6350933 29278293

[263] Doan KV Ko CM Kinyua AW Yang DJ Choi YH Oh IY. Gallic acid regulates body weight and glucose homeostasis through AMPK activation. Endocrinology. (2015) 156:157–68. 10.1210/en.2014-1354 25356824

[264] Huang DW Chang WC Yang HJ Wu JS Shen SC. Gallic acid alleviates hypertriglyceridemia and fat accumulation via modulating glycolysis and lipolysis pathways in perirenal adipose tissues of rats fed a high-fructose diet. Int J Mol Sci. (2018) 19:254. 10.3390/ijms19010254 29342975 PMC5796201

[265] Quezada-Fernandez P Trujillo-Quiros J Pascoe-Gonzalez S Trujillo-Rangel WA Cardona-Muller D Ramos-Becerra CG. Effect of green tea extract on arterial stiffness, lipid profile and sRAGE in patients with type 2 diabetes mellitus: a randomised, double-blind, placebo-controlled trial. Int J Food Sci Nutr. (2019) 70:977–85. 10.1080/09637486.2019.1589430 31084381

[266] Most J Timmers S Warnke I Jocken JW van Boekschoten M de Groot P Combined epigallocatechin-3-gallate and resveratrol supplementation for 12 wk increases mitochondrial capacity and fat oxidation, but not insulin sensitivity, in obese humans: a randomized controlled trial. Am J Clin Nutr. (2016) 104:215–27. 10.3945/ajcn.115.122937 27194304

[267] Mellor DD Sathyapalan T Kilpatrick ES Beckett S Atkin SL. High-cocoa polyphenol-rich chocolate improves HDL cholesterol in Type 2 diabetes patients. Diabet Med. (2010) 27:1318–21. 10.1111/j.1464-5491.2010.03108.x 20968113

[268] Lee YS Cha BY Choi SS Choi BK Yonezawa T Teruya T. Nobiletin improves obesity and insulin resistance in high-fat diet-induced obese mice. J Nutr Biochem. (2013) 24:156–62. 10.1016/j.jnutbio.2012.03.014 22898571

[269] Huong DT Takahashi Y Ide T. Activity and mRNA levels of enzymes involved in hepatic fatty acid oxidation in mice fed citrus flavonoids. Nutrition. (2006) 22:546–52. 10.1016/j.nut.2005.11.006 16483743

[270] Mulvihill EE Allister EM Sutherland BG Telford DE Sawyez CG Edwards JY. Naringenin prevents dyslipidemia, apolipoprotein B overproduction, and hyperinsulinemia in LDL receptor-null mice with diet-induced insulin resistance. Diabetes. (2009) 58:2198–210. 10.2337/db09-0634 19592617 PMC2750228

[271] Cho KW Kim YO Andrade JE Burgess JR Kim YC. Dietary naringenin increases hepatic peroxisome proliferators-activated receptor alpha protein expression and decreases plasma triglyceride and adiposity in rats. Eur J Nutr. (2011) 50:81–8. 10.1007/s00394-010-0117-8 20567977

[272] Goldwasser J Cohen PY Lin W Kitsberg D Balaguer P Polyak SJ. Naringenin inhibits the assembly and long-term production of infectious hepatitis C virus particles through a PPAR-mediated mechanism. J Hepatol. (2011) 55:963–71. 10.1016/j.jhep.2011.02.011 21354229 PMC3197749

[273] Ren B Qin W Wu F Wang S Pan C Wang L. Apigenin and naringenin regulate glucose and lipid metabolism, and ameliorate vascular dysfunction in type 2 diabetic rats. Eur J Pharmacol. (2016) 773:13–23. 10.1016/j.ejphar.2016.01.002 26801071

[274] Yang MY Chan KC Lee YJ Chang XZ Wu CH Wang CJ. Sechium edule shoot extracts and active components improve obesity and a fatty liver that involved reducing hepatic lipogenesis and adipogenesis in high-fat-diet-fed rats. J Agric Food Chem. (2015) 63:4587–96. 10.1021/acs.jafc.5b00346 25912298

[275] Zhang J Zhang H Deng X Zhang N Liu B Xin S. Baicalin attenuates non-alcoholic steatohepatitis by suppressing key regulators of lipid metabolism, inflammation and fibrosis in mice. Life Sci. (2018) 192:46–54. 10.1016/j.lfs.2017.11.027 29158052

[276] Guo HX Liu DH Ma Y Liu JF Wang Y Du ZY. Long-term baicalin administration ameliorates metabolic disorders and hepatic steatosis in rats given a high-fat diet. Acta Pharmacol Sin. (2009) 30:1505–12. 10.1038/aps.2009.150 19890358 PMC4003011

[277] Han M Gao H Ju P Gao MQ Yuan YP Chen XH. Hispidulin inhibits hepatocellular carcinoma growth and metastasis through AMPK and ERK signaling mediated activation of PPARgamma. Biomed Pharmacother. (2018) 103:272–83. 10.1016/j.biopha.2018.04.014 29656183

[278] Chen S Zhao X Wan J Ran L Qin Y Wang X. Dihydromyricetin improves glucose and lipid metabolism and exerts anti-inflammatory effects in nonalcoholic fatty liver disease: a randomized controlled trial. Pharmacol Res. (2015) 99:74–81. 10.1016/j.phrs.2015.05.009 26032587

[279] Chang X Wang Z Zhang J Yan H Bian H Xia M. Lipid profiling of the therapeutic effects of berberine in patients with nonalcoholic fatty liver disease. J Transl Med. (2016) 14:266. 10.1186/s12967-016-0982-x 27629750 PMC5024486

[280] Kasala ER Bodduluru LN Barua CC Gogoi R. Antioxidant and antitumor efficacy of Luteolin, a dietary flavone on benzo(a)pyrene-induced experimental lung carcinogenesis. Biomed Pharmacother. (2016) 82:568–77. 10.1016/j.biopha.2016.05.042 27470398

[281] Chen S Jiang H Wu X Fang J. therapeutic effects of quercetin on inflammation, obesity, and type 2 diabetes. Mediators Inflamm. (2016) 2016:9340637. 10.1155/2016/9340637 28003714 PMC5149671

[282] Porras D Nistal E Martinez-Florez S Pisonero-Vaquero S Olcoz JL Jover R. Protective effect of quercetin on high-fat diet-induced non-alcoholic fatty liver disease in mice is mediated by modulating intestinal microbiota imbalance and related gut-liver axis activation. Free Radic Biol Med. (2017) 102:188–202. 10.1016/j.freeradbiomed.2016.11.037 27890642

[283] Shang J Chen LL Xiao FX Sun H Ding HC Xiao H. Resveratrol improves non-alcoholic fatty liver disease by activating AMP-activated protein kinase. Acta Pharmacol Sin. (2008) 29:698–706. 10.1111/j.1745-7254.2008.00807.x 18501116

[284] Aguirre L Portillo MP Hijona E Bujanda L. Effects of resveratrol and other polyphenols in hepatic steatosis. World J Gastroenterol. (2014) 20:7366–80. 10.3748/wjg.v20.i23.7366 24966607 PMC4064082

[285] Andrade JM Paraiso AF de Oliveira MV Martins AM Neto JF Guimaraes AL. Resveratrol attenuates hepatic steatosis in high-fat fed mice by decreasing lipogenesis and inflammation. Nutrition. (2014) 30:915–9. 10.1016/j.nut.2013.11.016 24985011

[286] Ejaz A Wu D Kwan P Meydani M. Curcumin inhibits adipogenesis in 3T3-L1 adipocytes and angiogenesis and obesity in C57/BL mice. J Nutr. (2009) 139:919–25. 10.3945/jn.108.100966 19297423

[287] Colacino JA McDermott SP Sartor MA Wicha MS Rozek LS. Transcriptomic profiling of curcumin-treated human breast stem cells identifies a role for stearoyl-coa desaturase in breast cancer prevention. Breast Cancer Res Treat. (2016) 158:29–41. 10.1007/s10549-016-3854-4 27306423 PMC5831404

[288] Polce SA Burke C Franca LM Kramer B de Andrade Paes AM Carrillo-Sepulveda MA. Ellagic acid alleviates hepatic oxidative stress and insulin resistance in diabetic female rats. Nutrients. (2018) 10:531. 10.3390/nu10050531 29693586 PMC5986411

[289] Moore J Yousef M Tsiani E. Anticancer effects of rosemary (rosmarinus officinalis l.) extract and rosemary extract polyphenols. Nutrients. (2016) 8:731. 10.3390/nu8110731 27869665 PMC5133115

[290] Naimi M Vlavcheski F Shamshoum H Tsiani E. Rosemary extract as a potential anti-hyperglycemic agent: current evidence and future perspectives. Nutrients. (2017) 9:968. 10.3390/nu9090968 28862678 PMC5622728

[291] Lodi A Saha A Lu X Wang B Sentandreu E Collins M Combinatorial treatment with natural compounds in prostate cancer inhibits prostate tumor growth and leads to key modulations of cancer cell metabolism. NPJ Precis Oncol. (2017) 1:1–12. 10.1038/s41698-017-0024-z 29202102 PMC5705091

[292] Park KH Shin HJ Song YB Hyun HC Cho HJ Ham HS. Possible role of ginsenoside Rb1 on regulation of rat liver triglycerides. Biol Pharm Bull. (2002) 25:457–60. 10.1248/bpb.25.457 11995924

[293] Shen L Xiong Y Wang DQ Howles P Basford JE Wang J. Ginsenoside Rb1 reduces fatty liver by activating AMP-activated protein kinase in obese rats. J Lipid Res. (2013) 54:1430–8. 10.1194/jlr.M035907 23434611 PMC3622335

[294] Qiao L Zhang X Liu M Liu X Dong M Cheng J Ginsenoside Rb1 enhances atherosclerotic plaque stability by improving autophagy and lipid metabolism in macrophage foam cells. Front Pharmacol. (2017) 8:727 10.3389/fphar.2017.00727 29114222 PMC5660703

[295] Colman-Martinez M Martinez-Huelamo M Valderas-Martinez P Arranz-Martinez S Almanza-Aguilera E Corella D. Trans-Lycopene from tomato juice attenuates inflammatory biomarkers in human plasma samples: an intervention trial. Mol Nutr Food Res. (2017) 61. 10.1002/mnfr.201600993 28688174

[296] Brandhorst S Choi IY Wei M Cheng CW Sedrakyan S Navarrete G. A periodic diet that mimics fasting promotes multi-system regeneration, enhanced cognitive performance, and healthspan. Cell Metab. (2015) 22:86–99. 10.1016/j.cmet.2015.05.012 26094889 PMC4509734

